# Machine learning for detection of stenoses and aneurysms: application in a physiologically realistic virtual patient database

**DOI:** 10.1007/s10237-021-01497-7

**Published:** 2021-07-31

**Authors:** G. Jones, J. Parr, P. Nithiarasu, S. Pant

**Affiliations:** 1 Faculty of Science and Engineering, Swansea University, Swansea, UK; 2 McLaren Technology Centre, Woking, UK

**Keywords:** Virtual patients, Stenosis, Aneurysm, Pulse wave haemodynamics, Screening, Machine learning

## Abstract

This study presents an application of machine learning (ML) methods for detecting the presence of stenoses and aneurysms in the human arterial system. Four major forms of arterial disease—carotid artery stenosis (CAS), subclavian artery stenosis (SAS), peripheral arterial disease (PAD), and abdominal aortic aneurysms (AAA)—are considered. The ML methods are trained and tested on a physiologically realistic virtual patient database (VPD) containing 28,868 healthy subjects, adapted from the authors previous work and augmented to include disease. It is found that the tree-based methods of Random Forest and Gradient Boosting outperform other approaches. The performance of ML methods is quantified through the $F_1$ score and computation of sensitivities and specificities. When using six haemodynamic measurements (pressure in the common carotid, brachial, and radial arteries; and flow-rate in the common carotid, brachial, and femoral arteries), it is found that maximum $F_1$ scores larger than 0.9 are achieved for CAS and PAD, larger than 0.85 for SAS, and larger than 0.98 for both low- and high-severity AAAs. Corresponding sensitivities and specificities are larger than 90% for CAS and PAD, larger than 85% for SAS, and larger than 98% for both low- and high-severity AAAs. When reducing the number of measurements, performance is degraded by less than 5% when three measurements are used, and less than 10% when only two measurements are used for classification. For AAA, it is shown that $F_1$ scores larger than 0.85 and corresponding sensitivities and specificities larger than 85% are achievable when using only a single measurement. The results are encouraging to pursue AAA monitoring and screening through wearable devices which can reliably measure pressure or flow-rates.

## Introduction

Two of the most common forms of arterial disease are stenosis, narrowing of an arterial vessel, and aneurysm, an increase in the area of a vessel. They are estimated to affect between 1 and 20% of the population (Fowkes et al. 2013; Shadman et al. 2004; Mathiesen et al. 2001; Li et al. 2013), and ruptured abdominal aortic aneurysms alone are estimated to cause 6000–8000 deaths per year in the United Kingdom (Darwood et al. 2012). Current methods for the detection of arterial disease are primarily based on direct imaging of the vessels, which can be expensive and hence prohibitive for large-scale screening. If arterial disease can be detected by easily acquirable pressure and flow-rate measurements at select locations within the arterial network, then large-scale screening may be facilitated.

It is likely that the indicative biomarkers of arterial disease in the pressure and flow-rate profiles consist of micro inter- and intra-measurement details. In the past, detection of arterial disease has been proposed through the analysis of waveforms in combination with mathematical models of pulse wave propagation, see for example Sazonov et al. (2017), Stergiopulos et al. (1992). This, however, requires specification or identification of patient-specific network parameters, which is not easy to perform, especially at large scales.

This study explores the use of machine learning (ML) methods for the detection of stenoses and aneurysms in order to facilitate large scale low-cost screening/diagnosis. A data-driven ML approach is adopted, which does not require specification of patient-specific parameters. Instead, such algorithms learn patterns and biomarkers from a labelled data set. ML has a history of being used for medical applications (Kononenko 2001). Classification algorithms have been shown to be able to predict the presence of irregularities in heart valves (Çomak et al. 2007), arrhythmia (Song et al. 2005), and sleep apnea (Khandoker et al. 2009) from recorded time domain data. Recently, a study reported the successful use of ML methods to estimate pulse wave velocity from radial pressure wave measurements (Jin et al. 2020). Automatic detection, segmentation, and classification of AAAs in CT images are presented in Hong and Sheikh (2016), while severity growth of AAAs is predicted from CT images in Jiang et al. (2020). A previous study (Chakshu et al. 2020) has applied deep-learning methods to AAA classification, using a synthetic data set created by varying seven parameters. In this study, accuracies of $\approx 99.9\%$ are reported for binary classification of AAA based on three pressure measurements. Furthermore, Wang et al. (2021) achieved a sensitivity of 86.8% and a specificity of 86.3% for early detection of AAA from the photoplethysmogram pulse waves—using a synthetic data set created by finding the mean and standard deviation of six cardiovascular properties for subjects of each age decade from 55 to 75 years, and then varying each property in combination with each other by ± 1 standard deviation from their age-specific mean values. These studies motivate the application of ML to detect arterial disease—both stenosis and aneurysms—using only pressure and flow-rate measurements at select locations in the arterial network. A previous proof-of-concept study (Jones et al. 2021c) showed promising results that ML classifiers can detect stenosis in a simple three vessel arterial network using only measurements of pressures and flow-rates. Here, these ideas are extended to a significantly larger, physiologically realistic, network of the human arterial system. All the ML methods are trained and tested on the virtual healthy subject database proposed in Jones et al. (2021a), which is augmented to introduce disease into the virtual subjects.

This study is organised as follows. It begins by briefly explaining the healthy VPD proposed in Jones et al. (2021a). Modifications to this database to create four different forms of arterial disease are presented next, along with the parameterisation of disease forms. This is followed by presentation of the ML methodology and metrics used for quantification of classification accuracies. Finally, these accuracies are assessed when using different combinations of pressure and flow-rate measurements, along with the analysis of patterns and behaviours observed in the ML classifiers.

## Methodology

The ML algorithms are trained and tested on a data set containing both healthy subjects and diseased patients.

### Healthy subjects

**Fig. 1:**
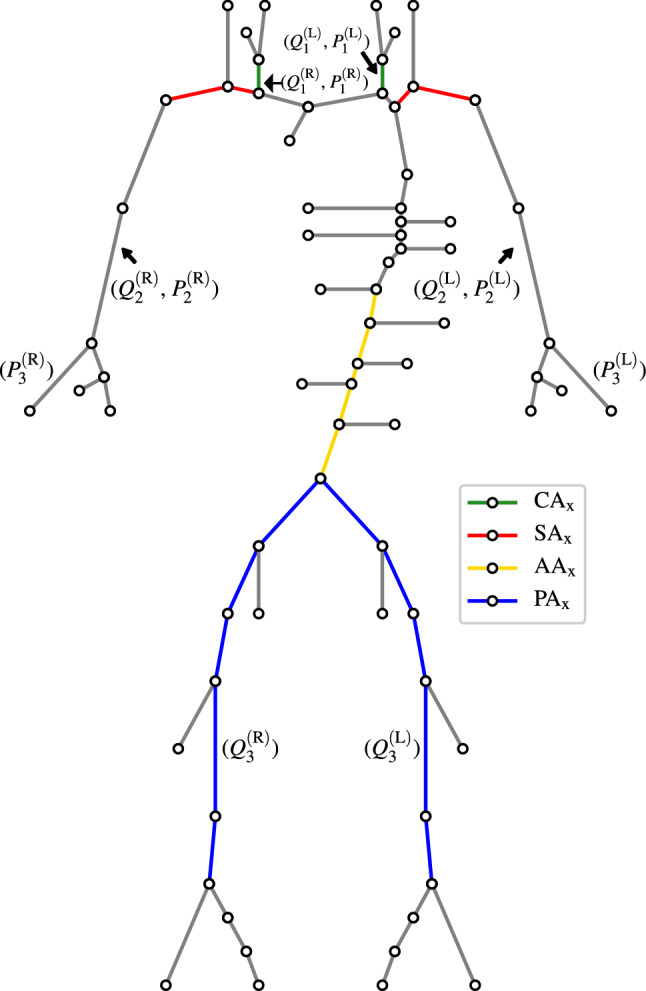
The connectivity of the arterial network, taken from Jones et al. (2021a). The location of the four forms of disease (see Sect. 2.2.1); and six pressure and flow-rate measurements (see Sect. 2.3) are highlighted

A physiologically realistic VPD containing healthy subjects is created in Jones et al. (2021a) and forms the starting point of this study. This database is available in Jones et al. (2021b). The arterial network contains 71 vessel segments and is shown in Fig. 1, along with the locations where disease occurs in high prevalence, and where measurements of pressure and flow-rate can potentially be acquired (Jones et al. 2021a). The healthy patient database of Jones et al. (2021a) contains 28,868 VPs and is referred as $\text {VPD}_{\text {H}}$. Disease is introduced into these healthy arterial networks as described next.

### Creation of unhealthy VPDs

#### Disease forms

The four most common forms of arterial disease are carotid artery stenosis (CAS), subclavian artery stenosis (SAS), peripheral arterial disease (PAD, a form of stenosis), and abdominal aortic aneurysm (AAA) (Jones et al. 2021a; Dyken et al. 1974; Kullo and Rooke 2016; Aboyans et al. 2010; Chen et al. 2013; Li et al. 2013). Their prevalence is restricted to the following vessels and shown in Fig. 1:

- *CAS* is assumed to only affect the common carotid arteries. For simplification and consistency of notation, these vessels are referred to as the *carotid artery chains* ($\hbox {CA}_{{\mathbf {x}}}$).
- *SAS* is assumed to affect the first and second subclavian segments. These two chains of vessels (one on the right and left side) are referred to as the *subclavian artery chains* ($\hbox {SA}_{{\mathbf {x}}}$).
- *PAD* is assumed to affect the common iliacs; external iliacs; first and second femoral segments; and the first popliteal segments. These chains are referred to as the *peripheral artery chains* ($\hbox {PA}_{{\mathbf {x}}}$).
- *AAA* is assumed to affect the first to forth abdominal aorta segment. This chain of vessels is referred to as the *abdominal aortic chain* ($\hbox {AA}_{{\mathbf {x}}}$).

It is assumed that each diseased VP has only one of the four forms of arterial disease. Four complementary databases corresponding to $\text {VPD}_{\text {H}}$ are constructed, each pertaining to one form of arterial disease. To create the diseased VPD corresponding to CAS, referred to as $\text {VPD}_{\text {CAS}}$, for every subject in $\text {VPD}_{\text {H}}$, disease is introduced in $\hbox {CA}_{\mathrm {x}}$ (i.e. the left or right carotid artery). This is achieved by taking the arterial network of a subject from $\hbox {VPD}_{\text {H}}$, artificially introducing a stenosis in $\hbox {CA}_{\mathrm {x}}$, and then using a one-dimensional pulse-wave propagation model—which has previously been widely employed, tested, and validated (Boileau et al. 2015; Formaggia et al. 2003; Alastruey et al. 2012; Olufsen et al. 2000; Reymond et al. 2009; Matthys et al. 2007)—to compute the pressure and flow-rate waveforms. Note that this model has also been used to study haemodynamics in both stenosis (Boileau et al. 2018; Carson et al. 2019; Jin and Alastruey 2021) and aneurysms (Sazonov et al. 2017; Chakshu et al. 2020; Jin and Alastruey 2021). The numerical implementation of the pulse-wave propagation model employed here is outlined in Jones et al. (2021a) and validated against a discontinuous Galerkin (DCG) scheme (Alastruey et al. 2012), which in turn has been successfully validated against a 3D model of blood-flow through stenosed arterial vessels (Boileau et al. 2018).

Thus, $\text {VPD}_{\text {CAS}}$ contains 28,868 VPs with CAS. Similarly, the databases corresponding to SAS, PAD, and AAA are created, and referred to as $\text {VPD}_{\text {SAS}}$, $\text {VPD}_{\text {PAD}}$, and $\text {VPD}_{\text {AAA}}$, respectively. The disease severities, locations, and shapes are varied randomly across these databases as described next.

#### Parameterisation of diseased vessels

**Fig. 2:**
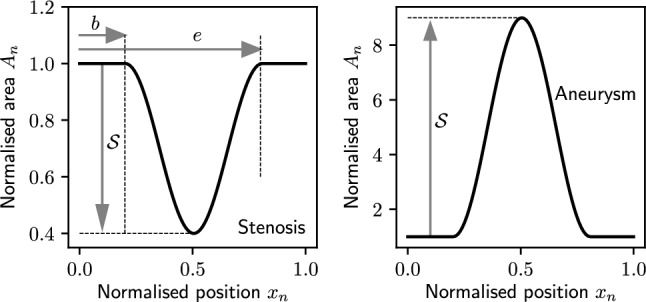
An example of a stenosis of severity 0.6 and aneurysm of severity 8.0 is shown. These disease profiles are created with a start location of 0.2 and an end location of 0.8

The severity of stenoses (percentage reduction in area) is varied between 50 and 95%. The lower 50% limit is set for the stenoses to be haemodynamically significant (Aboyans et al. 2010; Subramanian et al. 2005) and the upper limit of 95% reflects near total occlusion. For aneurysms, based on (Ernst 1993) and (Davis et al. 2013), an allowable range of AAA severities of 4cm–6cm diameters is chosen. This corresponds to a cross-sectional area variation of $12.56\text { cm}^2$–$28.27\text { cm}^2$. With the abdominal aortic area in the reference network (Jones et al. 2021a) between 1.76 and $1.09\text { cm}^2$, the corresponding AAA severities are set to vary between 713% (12.56/1.76) and 2,593% (28.27/1.09). With the above ranges, parameterisation of area increase/reduction proposed in Jones et al. (2021c) is adopted, see Fig. 2. For a chain of diseased vessels ($\hbox {CA}_{\text {x}}$, $\hbox {SA}_{\text {x}}$, $\hbox {PA}_{\text {x}}$, or $\hbox {AA}_{\text {x}}$), the normalised area $A_n$ as a function of the normalised x-coordinate, $x_n$, is represented as:

1 $$\begin{aligned} A_{n}\!=\! {\left\{ \begin{array}{ll} \bigg (1\! \mp \! \dfrac{{\mathcal {S}}}{2} \bigg ) \pm \dfrac{S}{2} \cos \left( \dfrac{2 (x_n-b) \pi }{e-b}\right) &{} \text {for } b\le x_n \le e \\ 1 &{} \text {otherwise} \end{array}\right. } \end{aligned}$$

where ${\mathcal {S}}$ represents the severity, *b* represents the normalised starting location of the disease in the vessel chain, *e* represents the normalised end location, $A_n$ is normalised with respect to the healthy version of the vessel in $\hbox {VPD}_{\text {H}}$, and ± creates an aneurysm or stenosis, respectively. In $\hbox {CA}_{\text {x}}$, $\hbox {SA}_{\text {x}}$, and $\hbox {PA}_{\text {x}}$, the left and right side vessels are chosen with equal probability.

The disease severity ${\mathcal {S}}$, start location *b*, and end location *e* are assigned uniform distributions based on physical considerations. To sample values for these parameters, a fourth parameter, the reference location of the disease (represented by *r*) is introduced. This is included to impose a minimum length of 10% of the chain length on the disease profiles. Thus, the parameters for disease are sampled sequentially from uniform distributions within the following bounds:

2 $$\begin{aligned} \text {Bounds:} {\left\{ \begin{array}{ll} 0.2 \le r \le 0.8,\\ 0.1 \le b \le r-0.05, \\ r+0.05 \le e \le 0.9,\\ {\left\{ \begin{array}{ll} 0.5 \le {\mathcal {S}} \le 0.95 &\quad\text {for stenoses,}\\ 7.13 \le {\mathcal {S}} \le 25.93 &\quad \text {for aneurysms.}\\ \end{array}\right. } \end{array}\right. } \end{aligned}$$

**Fig. 3:**
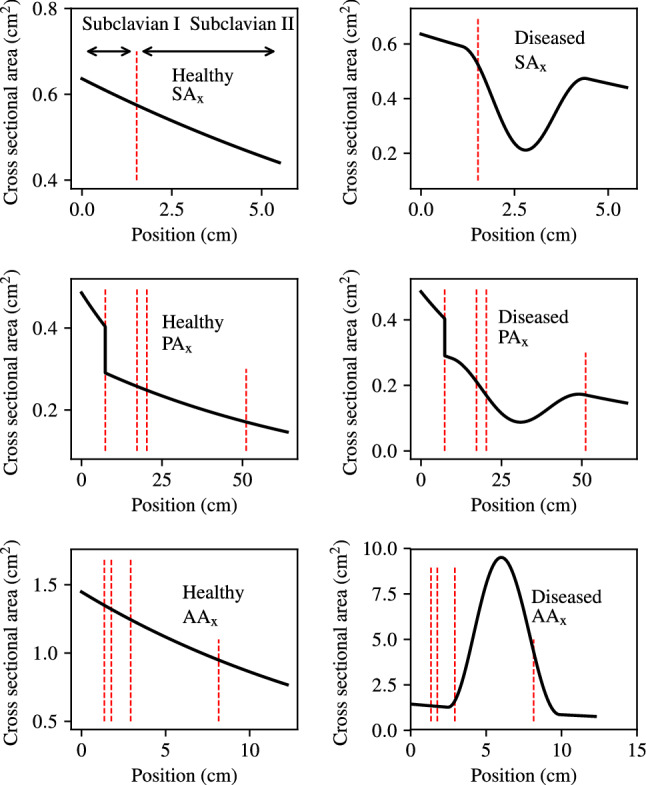
Examples of healthy and diseased $\hbox {SA}_{\text {x}}$, $\hbox {PA}_{\text {x}}$, and $\hbox {AA}_{\text {x}}$ area profiles. The geometrical boundaries between vessel segments that form the chains are indicated by red dashed lines

Based on the above parameterisation, examples of healthy and diseased $\hbox {SA}_{\text {x}}$, $\hbox {PA}_{\text {x}}$, and $\hbox {AA}_{\text {x}}$ area profiles are shown in the left and right columns of Fig. 3, respectively.

### Measurements

A review of potential measurements that can be acquired in the network is presented in Jones et al. (2021a). Based on this, the locations at which time-varying pressure and flow-rate measurements can be acquired are shown in Fig. 1 and described below.

- *Pressure in the carotid and radial arteries* measured using applanation tonometry (Adji et al. 2006; O’rourke 2015). To simplify annotation and description, the right and left carotid artery pressures are referred as $P_1^{\text {(R)}}$ and $P_1^{\text {(L)}}$, respectively. Similarly, the radial artery pressures are referred to $P_3^{\text {(R)}}$ and $P_3^{\text {(L)}}$, respectively.
- *Pressure in the brachial arteries* estimated through reconstruction of finger arterial pressure (Guelen et al. 2008). The right and left brachial artery pressures are referred to as $P_2^{\text {(R)}}$ and $P_2^{\text {(L)}}$ , respectively.
- *Flow-rate in the carotid, brachial, and femoral arteries* measured using Doppler ultrasound (Byström et al. 1998; Oglat et al. 2018; Radegran 1997). The right and left carotid artery, brachial, and femoral flow-rates are referred to as $Q_1^{\text {(R)}}$, $Q_1^{\text {(L)}}$; $Q_2^{\text {(R)}}$, $Q_2^{\text {(L)}}$; and $Q_3^{\text {(R)}}$, $Q_3^{\text {(L)}}$, respectively.

#### Provision of measurements to ML classifiers

Unless specified otherwise, the measurements to ML classifiers are bilateral, *i.e.* when $Q_1$ is specified it is implied that both right and left carotid flow-rates are used:

3 $$\begin{aligned} Q_1 = \{Q_1^{\text {(R)}}, Q_1^{\text {(L)}}\}. \end{aligned}$$

There are, therefore, a total of by six bilateral measurements available: three pressure and three flow-rates. To reduce the dimensionality required to describe each pressure or flow-rate measurement, the periodic profiles are described through a Fourier series (FS) representation:

4 $$\begin{aligned} u(t)=\sum _{n=0}^N a_n \sin (n \omega t) + b_n \cos (n \omega t), \end{aligned}$$

where *u* represents any pressure or flow-rate profile; $a_n$ and $b_n$ represent the $n{\text {th}}$ sine and cosine FS coefficients, respectively; *N* represents the truncation order; and $\omega ={2 \pi }/{T}$, with *T* as the time period of the cardiac cycle. It is found in Jones et al. (2021c) that haemodynamic profiles can be described by a FS truncated at $N=5$. Thus, each individual measurement is described by 11 FS coefficients, and each bilateral measurement by 22 FS coefficients.

### Machine learning classifiers

A model mapping a vector of input measurements, $\varvec{x}$, to a discrete output classification, *y*, can be described as:

5 $$\begin{aligned} y = m(\varvec{x}) \quad y \in \{{\mathcal {C}}^{(1)}, {\mathcal {C}}^{(2)}\}, \end{aligned}$$

where ${\mathcal {C}}^{(j)}$ represents the $j{\text {th}}$ possible classification. In the context of this study, the measured inputs, $\varvec{x}$, represent the FS coefficients of a user defined combination of the haemodynamic measurements $\{Q_1$, $Q_2$, $Q_3$, $P_1$, $P_2$, $P_3\}$ (see Sect. 2.3.1) taken from VPs, and the output classification represents the corresponding health of those VPs : ${\mathcal {C}}^{(1)}$= ‘healthy’ and ${\mathcal {C}}^{(2)}$= ‘diseased’. To account for large differences in magnitudes of the components of $\varvec{x}$, they are individually transformed with the Z-score standardisation method (Mohamad and Usman 2013) to have zero-mean and unit variance.

As previously stated, it assumed that in a patient disease is limited to only one of the four forms. As a first exploratory study, the ML classifiers are created for each form independently. All classifiers are therefore binary (see Jones et al. 2021c), *i.e.* four independent classifiers are trained to predict the following questions independently: *“Does a VP belong to* $\text {VPD}_{\text {H}}$ *or* $\text {VPD}_x$”, where *x* can be either CAS, SAS, PAD, or AAA.

#### Training and test sets

Each VP in $\text {VPD}_{\text {CAS}}$, $\text {VPD}_{\text {SAS}}$, $\text {VPD}_{\text {PAD}}$, and $\text {VPD}_{\text {AAA}}$ shares an identical underlying arterial network, apart from the diseased chain, with the corresponding healthy subject in $\hbox {VPD}_{\text {H}}$. It is, therefore, important to ensure that the same subset of VPs is not included in the both healthy and diseased data sets used for ML classifiers. As each form of disease is mutually exclusive, four independent training and test sets, each corresponding to one form of the disease, are constructed in the following three stages:

- *Step 1:* Half of the available VPs are randomly selected from $\text {VPD}_{\text {H}}$ for inclusion within the ML data set; this is referred to as $\text {VPD}_{\text {H-ML}}$. The unhealthy VPs corresponding to the remaining unused half are taken from the appropriate unhealthy VPD ($\text {VPD}_{\text {CAS}}$, $\text {VPD}_{\text {SAS}}$, $\text {VPD}_{\text {PAD}}$, or $\text {VPD}_{\text {AAA}}$) and incorporated into the ML data set. These data sets are referred to as $\text {VPD}_{\text {CAS-ML}}$, $\text {VPD}_{\text {SAS-ML}}$, $\text {VPD}_{\text {PAD-ML}}$, or $\text {VPD}_{\text {AAA-ML}}$.
- *Step 2:* The data sets of Step 1 are combined to create four complete data sets each containing 50% healthy and 50%, unhealthy VPs:
  1. $$\text {VPD}_{\text {H-ML}}\cup \text {VPD}_{\text {CAS-ML}}$$
  2. $$\text {VPD}_{\text {H-ML}}\cup \text {VPD}_{\text {SAS-ML}}$$
  3. $$\text {VPD}_{\text {H-ML}}\cup \text {VPD}_{\text {PAD-ML}}$$
  4. $$\text {VPD}_{\text {H-ML}}\cup \text {VPD}_{\text {AAA-ML}}$$
- *Step 3:* The four data sets of Step 2 are randomly split into a training set containing 2/3 of all the VPs in the data set, and a test set containing 1/3 of all the VPs.

The performance of all ML classifiers is evaluated using a fivefold validation. For each fold, the same data set from Step 2 is used, but different subsets are sampled in Step 3 for training and testing.

#### ML methods

The purpose of this study is to perform an initial exploratory investigation into the possibility of using ML classifiers to detect different forms of arterial disease. Focus is, therefore, on uncovering patterns and behaviours—such as which haemodynamic measurements are particularly informative—rather than optimisation to achieve increasingly higher accuracies. With consideration for this objective, it is not feasible to perform extensive optimisation and analysis on every single ML classifier trained and tested. Thus, the ML methods used are chosen based on their “robustness”—*i.e.* minimal sensitivity to the hyper-parameters and minimal susceptibility to problems such as overfitting—relative to more complex deep learning methods. Five different ML methods are employed. These five methods are random forest, gradient boosting, naive Bayes’, support vector machine, and logistic regression. These methods encompass a range of probabilistic and non-probabilistic applications of different modelling approaches, see Table 1, while fulfilling the aforementioned characteristics. Along with these five ML methods, one deep learning method is also employed for comparison. This method is multi-layer perceptron. It is *a priori* expected that multi-layer perceptron classifiers will not perform to their full potential in this study, as they are more reliant on complex hyper-parameter optimisation and monitoring for overfitting than the five ML methods. The use of multi-layer perceptrons will, however, provide some, albeit limited, comparison of ML and deep learning methods. Since standard versions and implementations of these methods are employed without any modifications, methodological details of these methods are not presented in this study. Instead, the reader is referred to the following references for methodological details:

1. Random Forest (RF) (Liaw and Wiener 2002; Breiman 2001)
2. Gradient Boosting (GB) (Friedman 2001; Elith et al. 2008)
3. Naive Bayes’ (NB) (Rish et al. 2001b, a)
4. Support Vector Machine (SVM) (Kecman 2005)
5. Logistic Regression (LR) (Sperandei 2014; Hilbe 2009; Jones et al. 2021c)
6. Multi-layer Perceptron (MLP) (Murtagh 1991)

All implementations of the above algorithms in the Python package Scikit-learn (Pedregosa et al. 2011) are used. Some of these methods require optimisation of the hyper-parameters. This is described after presenting performance quantification metrics in the next section.

**Table 1:** The four different modelling approaches and how each classification method aligns with these approaches

Modelling approach	Non-probabilistic	Probabilistic
Tree-based	RF	GB
Kernel-based	SVM	
Bayesian		NB
Neuron-based		LR, MLP

#### Quantification of results

**Fig. 4:**
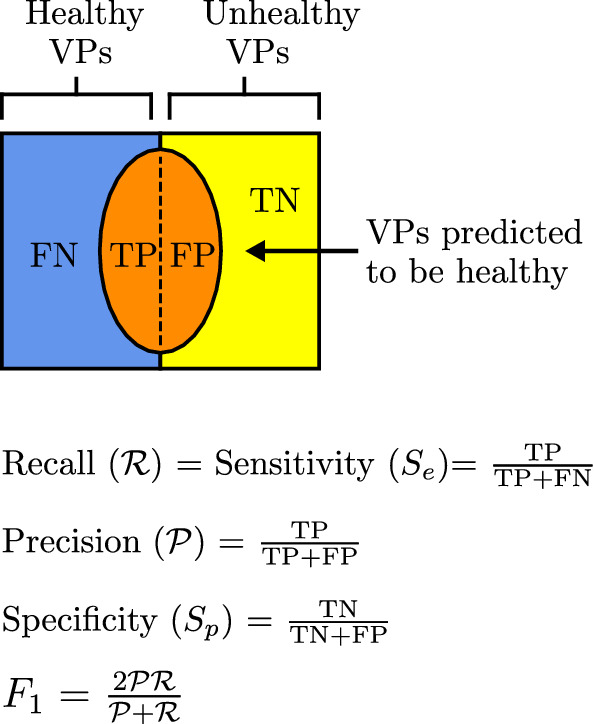
The relationship between sensitivity, specificity, recall, and precision. TP: True Positive, representing VPs belonging to a classification correctly identified; FN: False Negative, representing VPs belonging to a classification incorrectly identified: FP: False Positive, representing VPs not belonging to a classification incorrectly identified; and TN: True Negative, representing VPs not belonging to a classification correctly identified

Classifier performance is assessed by two metrics: *sensitivity* and *specificity* in combination; and the $F_1$ score. Figure 4 shows the definition of sensitivity, specificity, and $F_1$ score, along with the related concepts of *precision* and *recall* commonly used in the assessment of classifiers. It is desirable to have both sensitivities and specificities to be high. Similarly, a higher $F_1$ score is desirable. Since the $F_1$ score is a single scalar metric that balances both precision and recall, it is a good metric to compare classifiers when tuning the hyper-parameters of ML algorithms. For a discussion on these metrics and their relevance, please refer to Jones et al. (2021c).

### Hyper-parameter optimisation

The architecture of LR, NB, and SVM classifiers can all be considered to be problem independent. While these three algorithms are able to undergo varying levels of problem specific optimisation, the underlying structure of the classifier usually does not change. The architectures of RF, MLP, and GB classifiers, however, are dependent on the specific problem. The architecture choices for the classifiers and associated hyper-parameter optimisation are described next. For all six methods, all hyper-parameters that are neither optimised nor specified in the text are set to their default values within Scikit-learn (Pedregosa et al. 2011).

#### LR, SVM, and NB

For LR, the ‘LIBLINEAR’ solver offered by the Scikit-learn (Pedregosa et al. 2011) package is chosen. In the case of SVM, a kernel is typically chosen to map the input measurements to a higher order feature space (Jakkula 2006). All SVM classifiers use a radial basis function kernel (Scholkopf et al. 1997), with the Scikit-learn hyper-parameter ‘gamma’ set to ‘scale’. In the case of NB, the distribution of input measurements across the data set is chosen to be normal (Murphy et al. 2006).

#### Random Forest

In the case of RF, the number of trees in the ensemble and the maximum depth of each tree is optimised. Other hyper-parameters that can be tuned include the minimum number of data points allowed in a leaf node, and the maximum number of different features considered for splitting each node. However, the effect of these is not investigated here. To optimise the two hyper-parameters, a grid search is carried out. A grid is constructed by discretising the possible number of trees within the ensemble between 10 and 400 at intervals of 10, and the possible depth of each tree between 20 and 200 at intervals of 10. RF classifiers are trained for every combination with all six pressure and flow-rate measurements (see Sect. 2.3.1) across all the four forms of arterial disease. The hyper-parameters describing the architecture that produces the highest $F_1$ score are found for each form of disease, and this combination of hyper-parameters is then chosen for all subsequent classifiers. The optimal hyper-parameters for each of the four forms of disease are shown in Table 2, along with the $F_1$ score achieved by each.

**Table 2:** The hyper-parameters describing the architecture of the RF classifiers that produce the highest $F_1$ scores, when using all six pressure and flow-rate measurements

Disease	Trees	Depth	${F}_1$
CAS	100	80	0.8878
SAS	150	80	0.8292
PAD	100	100	0.8935
AAA	100	50	0.9912

It is unlikely that a single architecture will consistently produce the best results when varying the combination of input measurements. In this study, re-optimisation of the hyper-parameters when varying the input measurement combination is not performed, to minimise computational cost. It is found that when using all six pressure and flow-rate measurements, the $F_1$ score produced is relatively insensitive to the hyper-parameters used. Thus, it is likely that a reasonable representation of the maximum achievable accuracy can be obtained for various input measurement combinations by a single architecture. It should be noted, however, that further improvements in classification accuracy may be possible with such re-optimisation.

#### Gradient Boosting

Similar to the RF architecture, the GB architecture is optimised by varying the number of trees within the ensemble and the maximum depth of each tree. Other hyper-parameters which may be varied, however, are not considered here, are the minimum number of data points allowed in a leaf node, the maximum number of different features considered for splitting each node, and the impact of each tree on the final outcome (*i.e.* the learning rate). A grid search is carried out to find the combination producing the highest $F_1$ score when using all the six input measurements. It is common for GB classifiers to use weaker, shallower decision trees (relative to RF classifiers) to deliberately create high bias and low variance (Hastie et al. 2009). The possible depth of each tree is, therefore, discretised between 2 and 20 at intervals of 1. As a high number of trees is not required to compensate for over fitting, contrary to the RF method, the possible number of trees within the ensemble is discretised between 10 and 100 at intervals of 10. The optimal hyper-parameters for each of the four forms of disease are shown in Table 3.

**Table 3:** The hyper-parameters describing the architecture of the GB classifiers that produce the highest $F_1$ scores, when using all six pressure and flow-rate measurements

Disease	Trees	Depth	${F}_1$
CAS	100	6	0.9343
SAS	100	7	0.8574
PAD	100	10	0.9187
AAA	80	7	0.9970

#### Multi-layer perceptron

As is common with deep learning methods, relative to ML methods, there are significantly more hyper-parameters which can be optimised for the MLP classifiers relative to Gradient Boosting or Random Forest. Examples of hyper-parameters that significantly affect the performance of an MLP classifier include batch-size, learning rate, activation functions, drop-out, and individual units per hidden layers. With consideration for the exploratory stance of this study, it is chosen to only optimise the number of neurons within each hidden layer and the number of hidden layers. For simplification, it is assumed that all the hidden layers contain an identical number of neurons. A logistic activation function is used for all the hidden layers. It is likely that this simplistic hyper-parameter optimisation will limit the accuracy of classification achieved by MLP classifiers.

Similar to the RF and GB methodology, the hyper-parameters that produce the highest $F_1$ score are found through a grid search. The number of neurons within each layer is discretised between 10 and 200 at intervals of 10, and the number of hidden layers is discretised between 1 and 6 at intervals of 1. The optimal hyper-parameters found for each of the four forms of disease are shown in Table 4. It shows that relative to RF and GB, there is less consistency in the maximum $F_1$ scores achieved by MLP—it classifies AAA and CAS to high levels of accuracies, but performs relatively poorly for SAS and PAD.

**Table 4:** The hyper-parameters describing the architecture of the MLP classifiers that produce the highest $F_1$ scores, when using all six pressure and flow-rate measurements

Disease	Neurons	Depth	${F}_1$
CAS	60	4	0.7785
SAS	190	2	0.6040
PAD	120	2	0.6681
AAA	30	2	0.9785

### Input measurement combination search

There are 63 possible combinations of input measurements that can be provided to a ML classifier from the six bilateral pressure and flow-rate measurements (see Sect. 2.3.1). A combination search is performed for each of the four forms of disease. For every combination of input measurements, all the six ML classification methods are trained, and then subsequently tested to quantify their performance. The average $F_1$ score, sensitivity, and specificity for each case across five folds are recorded. Combinations of interest are then further analysed.

### Overfitting and early stopping criterion

To assess any overfitting by the ML and deep-learning methods, the log loss costs across the training and test sets are recorded at each sequential iteration of the training process (up to the 200${\text {th}}$ iteration). At a low number of training iterations, both the training and test costs are expected to be high as the classifiers can neither fit the training data nor generalise to the test data. As the training process progresses, the training and test costs are both expected to decay before converging to stable values in the absence of overfitting. However, in the case of overfitting, while the training costs continue to decrease, after a minima in the test costs, overfitting results in successively increasing test costs. In such cases, an early stopping criterion (Prechelt 1998; Yao et al. 2007) is adopted to avoid overfitting. A third partition to the available data (the validation set) is introduced. The combined healthy and unhealthy data sets described in Sect. 2.4.1 are split so that the training set contains 50%, the validation set 25%, and the test set 25% of the available data. Classifiers are trained on the training set; however, stopping criterion is based on the log loss cost in the validation set. At each sequential iteration in the training process, the average log loss cost is computed across the validation set. If more than 75 iterations have been performed, and the improvement in the log loss cost across the validation set between two consecutive iterations is less than $1\times 10^{-3}$, training is stopped. The final classifier accuracy is assessed on the test set.

## Results and discussion

**Fig. 5:**
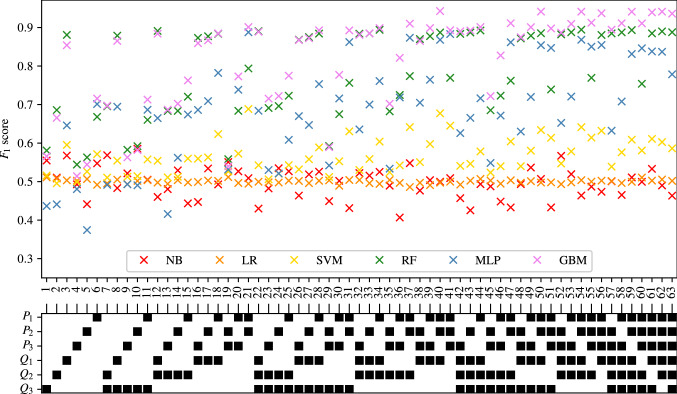
The $F_1$ scores achieved for CAS using each combination of bilateral input measurements are shown. Measurements included within each combination are highlighted with a black square

**Fig. 6:**
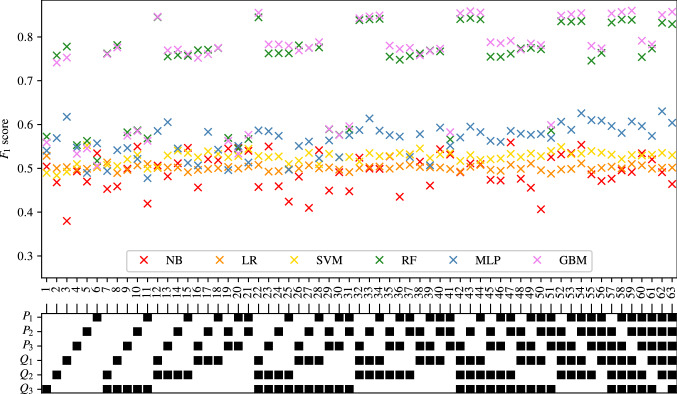
The $F_1$ scores achieved for SAS using each combination of bilateral input measurements are shown. Measurements included within each combination are highlighted with a black square

**Fig. 7:**
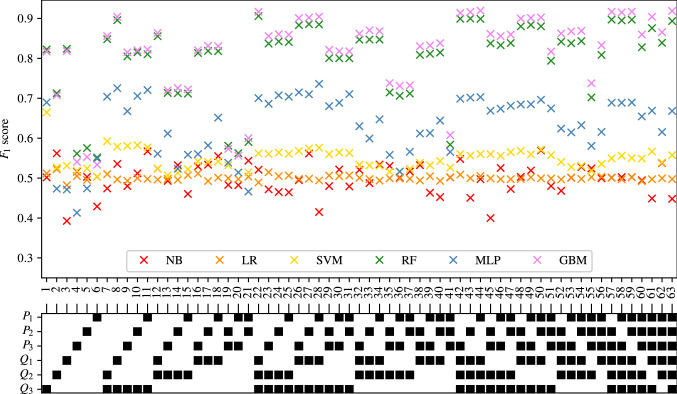
The $F_1$ scores achieved for PAD using each combination of bilateral input measurements are shown. Measurements included within each combination are highlighted with a black square

**Fig. 8:**
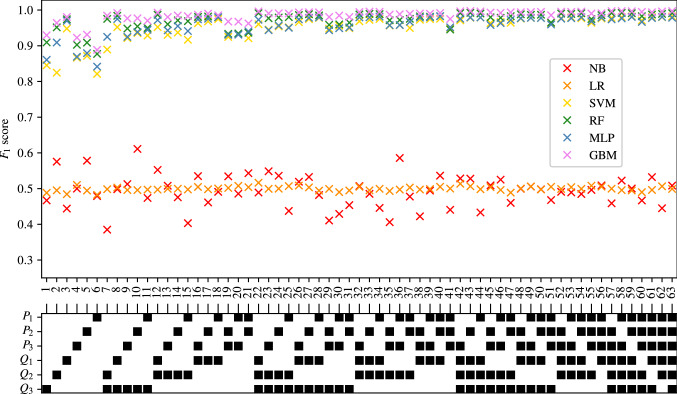
The $F_1$ scores achieved for AAA using each combination of bilateral input measurements are shown. Measurements included within each combination are highlighted with a black square

The full tables of results achieved for CAS, SAS, PAD, and AAA classification are shown in Appendices A, B, C and D, respectively. The $F_1$ score achieved by each ML method and combination of input measurements are visually shown for CAS, SAS, PAD, and AAA classification in Figs. 5, 6, 7, and 8, respectively. They show that for all forms of arterial disease, NB and LR classifiers consistently produce low accuracy. It has previously been shown in the PoC (Jones et al. 2021c) that the partition between the pressure and flow-rate profiles taken from healthy and stenosed patients is likely to be nonlinear. The fact that LR consistently produces low accuracy results supports this finding, as LR is the only linear classification method used. The finding that NB classifiers produce low accuracy classification is also consistent with the results of the PoC (Jones et al. 2021c), which found that the NB method is poorly suited to the problem of distinguishing between haemodynamic profiles. On the contrary, across all the four forms of disease, the tree-based methods (RF and GB) consistently produce high accuracy results. This finding is in contradiction to the finding in the PoC (Jones et al. 2021c) and is likely due to the inadequate architecture optimisation or because of the unsuitability of RF on a smaller network used in the PoC (Jones et al. 2021c). The fact that both RF and GB classifiers are producing high accuracy classification in this study suggests that not only are tree-based methods well suited to distinguishing between haemodynamic profiles, but also emphasises the importance of adequate architecture optimisation.

There is less consistency in the results achieved by SVM and MLP classifiers when detecting different forms of disease. SVM classifiers produce accuracies comparable with RF and GB classifiers in the case of AAA detection; however, low accuracy results for the three other forms of disease. MLP classifiers produce accuracies comparable with RF and GB classifiers in the case of CAS and AAA detection; however, relatively low accuracy results for SS and PAD classification. Overall, it is found that tree-based methods of RF and GB perform best, with GB performance slightly superior to that of RF. It is important to remember, however, that the results presented here do not necessarily capture the full potential of each method, and instead only reflect the accuracies achieved within the limitations of the simplistic hyper-parameter optimisation—a consideration particularly important for MLP.

### Measurement combinations

To investigate the importance of both the number of input measurements provided to the ML algorithms and the specific combination of measurements, the average $F_1$ scores achieved by all classifiers when providing only one, two, three, four, five, or six input measurements are found. In each case, the specific combinations that achieve the maximum and minimum $F_1$ scores are also recorded. These results for different forms of disease are presented next.

#### CAS classification

**Fig. 9:**
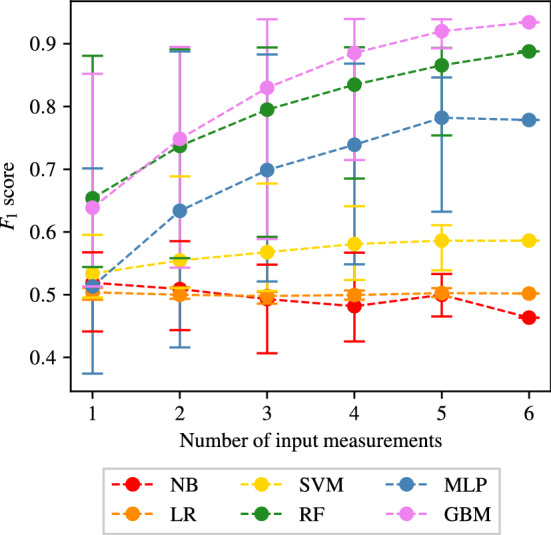
The average, maximum, and minimum $F_1$ score achieved by all classifiers trained using different numbers of input measurements are shown for carotid artery stenosis classification. The central markers represent the average score achieved, while the error bars indicate the upper and lower limits

The average, maximum and minimum $F_1$ score achieved when providing different number of input measurements for CAS classification are shown in Fig. 9.

It shows that NB and LR classifiers consistently produce an $F_1$ score of approximately 0.5, which is comparable to naive classification, *i.e.* randomly assigning the health of VPs with an equal probability to each outcome. SVM performs slightly better with $F_1$ scores averaging 0.5 – 0.6. The other three classification methods (RF, MLP, and GB) perform significantly better with $F_1$ scores generally averaging between 0.7 and 0.95 and showing a clear increase in the average $F_1$ score as the number of input measurements increases. While the average and minimum $F_1$ score achieved by RF and GB classifiers continuously increases, the maximum $F_1$ score achieved can be seen to quickly reach a plateau (at one input measurement for RF and three input measurements for GB). For a fixed number of measurements, the wide range of $F_1$ scores in Fig. 9 across all classifiers suggests that specific combinations of measurements may be more important than others for optimal classification. To explore this further, the combinations of input measurements that produce the highest $F_1$ scores and the corresponding accuracies when employing the RF and GB methods are shown in Table 5. Two observations are made from this table. First that for a fixed number of measurements, the best combinations are not identical for the two methods. For example, when two measurements are used the best combination for RF is ($Q_2$, $Q_1$), while the best combination for GB is ($P_2$, $P_1$). This suggests that the best combination of measurements is likely dependent on the particular ML method chosen. Second, some patterns stand out with respect to which measurements may be more informative than others. For example, across Table 5, $Q_1$ appears in 11 out of 12 combinations, and $P_1$ appears in 8 out of 12 combinations. This suggests that $Q_1$ is most informative about identifying the presence of CAS followed by $P_1$. Physiologically, this is not surprising as $Q_1$ and $P_1$ are flow-rates and pressures in the carotid arteries and the disease under consideration is carotid artery stenosis. It is encouraging that the ML methods are indeed placing more importance to the relevant physiological measurements. In fact, it is remarkable that RF and GB both achieve $F_1$ scores above 0.85 and sensitivities and specificities larger than 85% with only one measurement. Also notable is that these accuracies can be taken to beyond 93% (see GB row for 3 measurements in Table 5) when adding 2 more measurements as long as the additional two measurements are carefully chosen.

**Table 5:** The combinations of input measurements that produce the maximum $F_1$ scores when providing one to six input measurements and employing the RF and GB methods to detect CAS

Number of input measurements	Method	Combination	$F_1$ score	Sens.	Spec.
1	RF	($Q_1$)	0.8809	0.8704	0.8893
	GB	($Q_1$)	0.8521	0.8547	0.8502
2	RF	($Q_2$, $Q_1$)	0.8913	0.8765	0.9032
	GB	($P_2$, $P_1$)	0.8950	0.9026	0.8889
3	RF	($Q_2$, $Q_1$, $P_1$)	0.8941	0.8825	0.9035
	GB	($Q_1$, $P_2$, $P_1$)	0.9389	0.9433	0.9351
4	RF	($Q_2$, $Q_1$, $P_2$, $P_1$)	0.8944	0.8858	0.9015
	GB	($Q_3$, $Q_1$, $P_2$, $P_1$)	0.9395	0.9417	0.9376
5	RF	($Q_3$, $Q_2$, $Q_1$, $P_2$, $P_1$)	0.8934	0.8858	0.8996
	GB	($Q_2$, $Q_1$, $P_3$, $P_2$, $P_1$)	0.9391	0.9416	0.9370
6	RF	($Q_3$, $Q_2$, $Q_1$, $P_3$, $P_2$, $P_1$)	0.8878	0.8747	0.8984
	GB		0.9343	0.9364	0.9325

The corresponding sensitivities and specificities are also included

An interesting pattern to note is that while the average and minimum $F_1$ score achieved by MLP classifiers continuously increases in Fig. 9, the maximum $F_1$ score decreases beyond three input measurements. The maximum $F_1$ scores achieved by MLP classifiers, and the corresponding sensitivities and specificities, when using three to six input measurements are shown in Table 6. It shows that the decrease in $F_1$ scores is also accompanied by an associated decrease in both the sensitivities and specificities, as opposed to the balance between them (increase in sensitivity and decrease in specificity and vice versa). This behaviour is unusual as intuitively more input measurements should generally provide more information. This finding may suggest that MLP classifiers are able to extract maximum information from the haemodynamic profiles when using as little as three input measurements, and may be susceptible to over fitting when using more than three measurements, thereby leading to less generalisation capabilities and consequently decreased accuracies.

**Table 6:** The combinations of input measurements that produce the maximum $F_1$ scores when providing three to six input measurements and employing the MLP method to detect CAS

Number of input measurements	Combination	$F_1$ score	Sensitivity	Specificity
3	($P_3$, $P_2$, $P_1$)	0.8831	0.8731	0.8911
4	($Q_3$, $Q_1$, $P_2$, $P_1$)	0.8683	0.8538	0.8545
5	($Q_3$, $Q_2$, $P_3$, $P_2$, $P_1$)	0.8463	0.8308	0.8577
6	($Q_3$, $Q_2$, $Q_1$, $P_3$, $P_2$, $P_1$)	0.7785	0.7916	0.7703

The corresponding sensitivities and specificities are also included

**Fig. 10:**
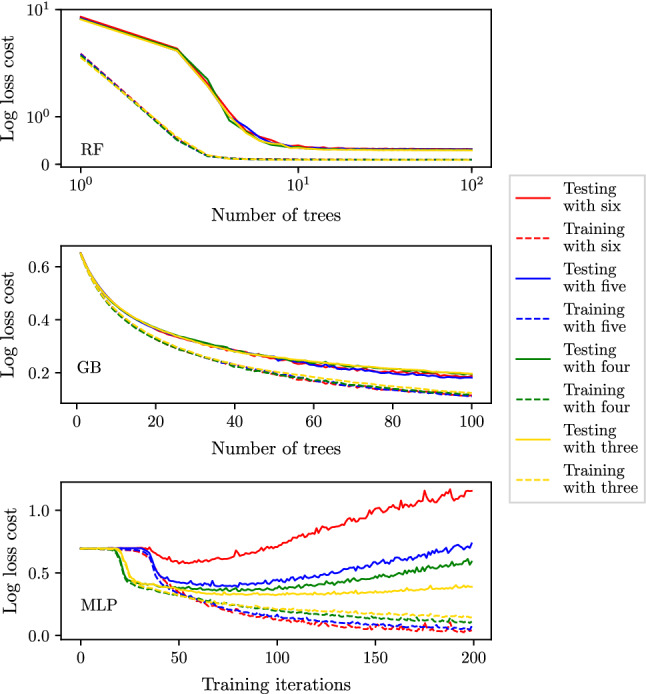
The average log loss cost across the training and test sets during the training process when using the combination of three to six input measurements that achieve highest accuracies for RF, GB, and MLP methods (Tables 5 and 6)

To investigate any overfitting, the log loss costs for the training and test sets during the training process are shown in Fig. 10 for the best measurement combinations identified by the MLP, GB, and RF method classifiers (Tables  5 and 6). It shows that the RF and GB methods show no signs of overfitting. However, for the MLP, while the three-measurement case also shows no overfitting, the cases with four, five, and six measurements show an increase in test costs beyond 50–100 training iterations, implying overfitting, the extent of which worsens as the number of measurements increases. Such behaviour for the MLP is also observed for SAS and PAD, and thus for the MLP method an early stopping criterion is adopted (see Sect. 2.7), the results of which are presented in Sect. 3.6.

#### SAS classification

**Fig. 11:**
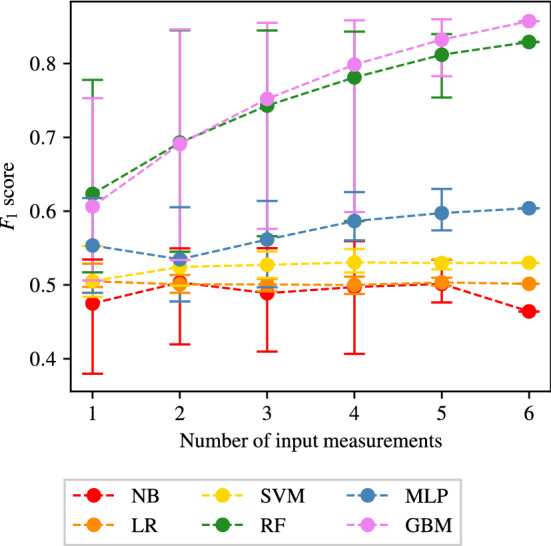
The average, maximum, and minimum $F_1$ score achieved by all classifiers trained using different numbers of input measurements are shown for SAS classification. The central markers represent the average score achieved, while the error bars indicate the upper and lower limits

The results of the analysis for SAS classification are shown in Fig. 11. As is seen in the case of CAS classification, Fig. 11 shows that NB, LR, and SVM classifiers consistently produce accuracies comparable to naive classification, irrespective of the number of input measurements used. A clear difference between Figs. 9 and 11 is the accuracy achieved by MLP classifiers. Compared to the CAS case, the MLP performance is further degraded for SAS, while still being better than NB, LR, and SVM, although only marginally. It is important to consider, however, that the MLP classifiers are experiencing overfitting, as highlighted in Sect. 3.1.1. Results with overfitting avoided by adopting an early stopping criterion are presented in Sect. 3.6.

A high degree of similarity can be seen between the behaviours of RF and GB classifiers for CAS and SAS. Figure 11 shows that the average and minimum $F_1$ score achieved by RF and GB classifiers continuously increases as the number of input measurements used increases. The maximum $F_1$ score achieved is seen to quickly reach an asymptotic limit (at three input measurements for both RF and GB classifiers). Peak $F_1$ score of approximately 0.85 is achieved by GB, along with sensitivities and specificities higher than 85%.

The combination of input measurements that produce the highest $F_1$ scores and the corresponding accuracies are shown in Table 7. It shows a higher degree of consistency between the best combinations for the two methods relative to the case for CAS, i.e. the best combinations are generally identical (or with minimal differences) between RF and GB. It also shows that $Q_1$ is particularly informative, with this measurement appearing in all of the best combinations. Physiologically this may be due to its proximity to the disease location.

**Table 7:** The combinations of input measurements that produce the maximum $F_1$ scores when providing one to six input measurements and employing the RF and GB methods to detect SAS

Number of input measurements	Method	Combination	$F_1$ score	Sens.	Spec.
1	RF	($Q_1$)	0.7779	0.7582	0.7905
	GB	($Q_1$)	0.7529	0.7224	0.7714
2	RF	($Q_2$, $Q_1$)	0.8450	0.8374	0.8507
	GB	($Q_2$, $Q_1$)	0.8461	0.8293	0.8585
3	RF	($Q_3$, $Q_2$, $Q_1$)	0.8447	0.8271	0.8576
	GB	($Q_3$, $Q_2$, $Q_1$)	0.8552	0.8453	0.8626
4	RF	($Q_3$, $Q_2$, $Q_1$, $P_2$)	0.8432	0.8303	0.8527
	GB	($Q_3$, $Q_2$, $Q_1$, $P_2$)	0.8585	0.8487	0.8660
5	RF	($Q_3$, $Q_2$, $Q_1$, $P_3$, $P_1$)	0.8399	0.8256	0.8504
	GB	($Q_3$, $Q_2$, $Q_1$, $P_2$, $P_1$)	0.8600	0.8525	0.8657
6	RF	(*Q*3, $Q_2$, $Q_1$, $P_3$, $P_2$, $P_1$)	0.8292	0.8102	0.8427
	GB		0.8574	0.8504	0.8627

The corresponding sensitivities and specificities are also included

#### PAD classification

**Fig. 12:**
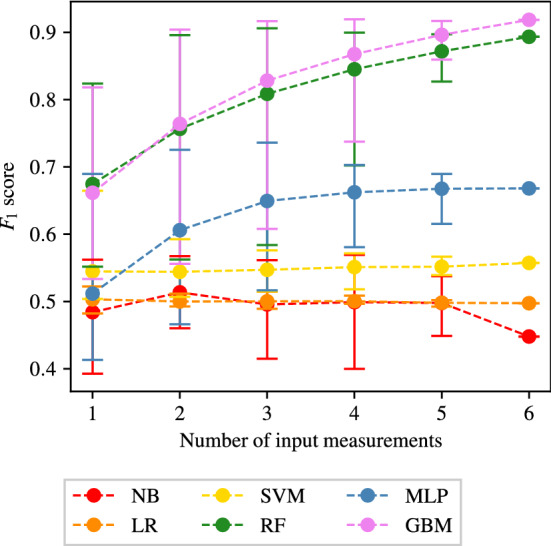
The average, maximum, and minimum $F_1$ score achieved by all classifiers trained using different numbers of input measurements are shown for PAD classification. The central markers represent the average score achieved, while the error bars indicate the upper and lower limits

The results for PAD classification are shown in Fig. 12. Comparing Figs. 11 and 12, a high degree of similarity is seen between the behaviours of SAS and PAD classification. As is previously seen for SAS classification, Fig. 12 shows that the NB, LR, and SVM methods are all consistently producing accuracies comparable to naive classification. While the MLP method performs slightly better than the naive classification, the accuracy still remains relatively low. High accuracy can be seen in Fig. 12 for the two tree-based methods of RF and GB. As has been previously seen for CAS and SAS, while the average and minimum $F_1$ score achieved by the RF and GB methods increases as the number of input measurements increases, the maximum $F_1$ score achieved quickly reaches an asymptotic limit (at 3 input measurements for both the RF and GB methods).

The combination of input measurements that produce the highest $F_1$ scores for PAD classification when employing the RF and GB methods are shown in Table 8. It not only shows good consistency between the combinations of input measurements that produce the highest $F_1$ scores when employing each of the two ML methods, but also good agreement with the combinations presented in Table 7. Very similar combinations of input measurements (with some minor differences) can be seen to produce the highest $F_1$ score when providing all numbers of input measurements. As has previously been observed in Tables 5 and 7, the input measurement $Q_1$ appears to be most informative, appearing in all the best scoring classifiers. Since the location of $Q_1$ is far from the location of disease, it is not obvious why this measurement is particularly informative of PAD.

**Table 8:** The combinations of input measurements that produce the maximum $F_1$ scores when providing one to six input measurements and employing the RF and GB methods to detect PAD

Number of input measurements	Method	Combination	$F_1$ score	Sens.	Spec.
1	RF	($Q_1$)	0.8240	0.8959	0.8320
	GB	($Q_1$)	0.8183	0.8126	0.8214
2	RF	($Q_3$, $Q_1$)	0.8140	0.8825	0.9068
	GB	($Q_3$, $Q_1$)	0.9041	0.8950	0.9117
3	RF	($Q_3$, $Q_2$, $Q_1$)	0.9061	0.8885	0.9208
	GB	($Q_3$, $Q_2$, $Q_1$)	0.9168	0.9055	0.9265
4	RF	($Q_3$, $Q_2$, $Q_1$, $P_2$)	0.8997	0.8868	0.9104
	GB	($Q_3$, $Q_2$, $Q_1$, $P_1$)	0.9196	0.9068	0.9306
5	RF	($Q_3$, $Q_2$, $Q_1$, $P_3$, $P_2$)	0.8971	0.8802	0.9110
	GB	($Q_3$, $Q_2$, $Q_1$, $P_2$, $P_1$)	0.9170	0.9041	0.9281
6	RF	(*Q*3, $Q_2$, $Q_1$, $P_3$, $P_2$, $P_1$)	0.8935	0.8813	0.9035
	GB		0.9187	0.9102	0.9261

The corresponding sensitivities and specificities are also included

#### AAA classification

**Fig. 13:**
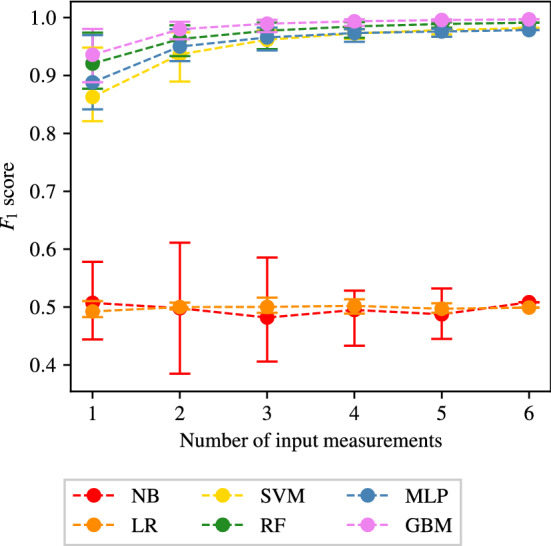
The average, maximum, and minimum $F_1$ score achieved by all classifiers trained using different numbers of input measurements are shown for AAA classification. The central markers represent the average score achieved, while the error bars indicate the upper and lower limits

The results for AAA classification are shown in Fig. 13. As has been previously seen for all of the three other forms of disease, the NB and LR classifiers consistently produce accuracies comparable to naive classification, irrespective of the number of input measurements used. The consistency of this finding (as seen in Figs. 9, 11, and 12) irrespective of the form of disease being classified, highlights both the importance of nonlinear partitions between healthy and unhealthy VPs and the unsuitability of the NB method for distinction between haemodynamic profiles.

In the case of AAA classification, the SVM, RF, MLP, and GB methods consistently produce good accuracies. Figure 13 shows that these methods produce high accuracies even with a single input measurement. While there is some increase in the average $F_1$ score as the number of input measurements increases, due to the very high initial average $F_1$ score achieved (when using a single input measurement) this increase is limited (as the $F_1$ score can not exceed 1). Two possible reasons of the higher accuracies in aneurysm classification relative to stenosis classification are:

- Aneurysms, owing to an increase in area as opposed to decrease in the area for stenoses, may actually produce more significant or consistent biomarkers in the pressure and flow-rate profiles. This hypothesis is supported by Low et al. (2012), which found that even low severity AAAs have a global impact on the pressure and flow-rate profiles.
- While the severities of aneurysms cannot be directly compared to severities of stenosis, it may be that the severity of aneurysms in $\text {VPD}_{\text {AAA}}$ is disproportionately large relative to the severities of stenoses. The significance of any indicative biomarkers introduced into pressure and flow-rate profiles is likely to be proportional to the severity of the change in area. This implies that the increase in vessel area of 712–2,593% in $\text {VPD}_{\text {AAA}}$ is perhaps on the extreme end of aneurysm severity, thereby making the classifications relatively easier. This is further explored in Sect. 3.4.

The combination of input measurements that produce the highest $F_1$ scores when providing one to six input measurements and employing the RF and GB methods are shown for AAA classification in Table 9. It shows that $F_1$ scores range from 0.97–0.997 and sensitivities and specificities range from 96.5% to 99.8%. Due to the high accuracies across all the number of measurements, the analysis of specific combinations is not very meaningful. However, the measurement $Q_1$ again appears in all the best combinations. It should also be noted that the high accuracies for AAA classification are also consistent with those reported in Chakshu et al. (2020)— where deep-learning methods are applied on a VPD created by varying seven network parameters, and classification accuracies of $\approx 99.9\%$ are reported—and (Wang et al. 2021)—where machine learning methods are applied on a VPD, and sensitivities and specificities of $\approx 86\%$ are reported.

Overall, the results show that the physiological changes to the waveforms induced by both stenosis and aneurysms (Stergiopulos et al. 1992; Low et al. 2012) are well captured by the data-driven machine learning methods.

**Table 9:** The combinations of input measurements that produce the maximum $F_1$ scores when providing one to six input measurements and employing the RF and GB methods to detect AAA

Number of input measurements	Method	Combination	$F_1$ score	Sens.	Spec.
1	RF	($Q_1$)	0.9741	0.9654	0.9825
	GB	($Q_1$)	0.9805	0.9799	0.9811
2	RF	($Q_2$, $Q_1$)	0.9868	0.9810	0.9926
	GB	($Q_2$, $Q_1$)	0.9928	0.9919	0.9938
3	RF	($Q_3$, $Q_2$, $Q_1$)	0.9912	0.9864	0.9961
	GB	($Q_3$, $Q_2$, $Q_1$)	0.9962	0.9954	0.9970
4	RF	($Q_3$, $Q_2$, $Q_1$, $P_2$)	0.9923	0.9879	0.9967
	GB	($Q_3$, $Q_2$, $Q_1$, $P_2$)	0.9972	0.9959	0.9986
5	RF	($Q_3$, $Q_2$, $Q_1$, $P_3$, $P_1$)	0.9920	0.9873	0.9967
	GB	($Q_3$, $Q_2$, $Q_1$, $P_3$, $P_2$)	0.9970	0.9959	0.9981
		($Q_3$, $Q_2$, $Q_1$, $P_3$, $P_1$)		0.9963	0.9978
6	RF	(*Q*3, $Q_2$, $Q_1$, $P_3$, $P_2$, $P_1$)	0.9912	0.9861	0.9964
	GB		0.9970	0.9959	0.9981

The corresponding sensitivities and specificities are also included

### Importance of carotid artery flow-rate

**Fig. 14:**
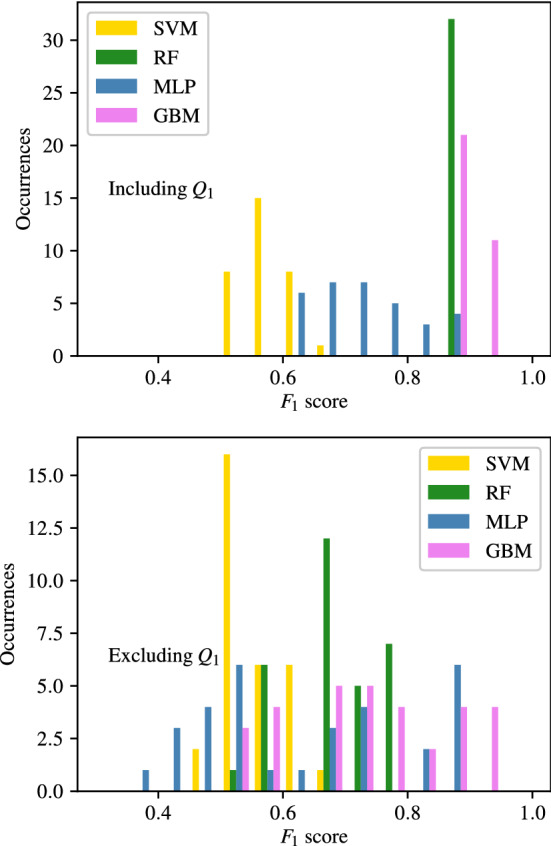
The histograms of the $F_1$ scores achieved for CAS classification are shown for all input measurement combinations that include $Q_1$ in the upper plot and exclude $Q_1$ in the lower plot

**Fig. 15:**
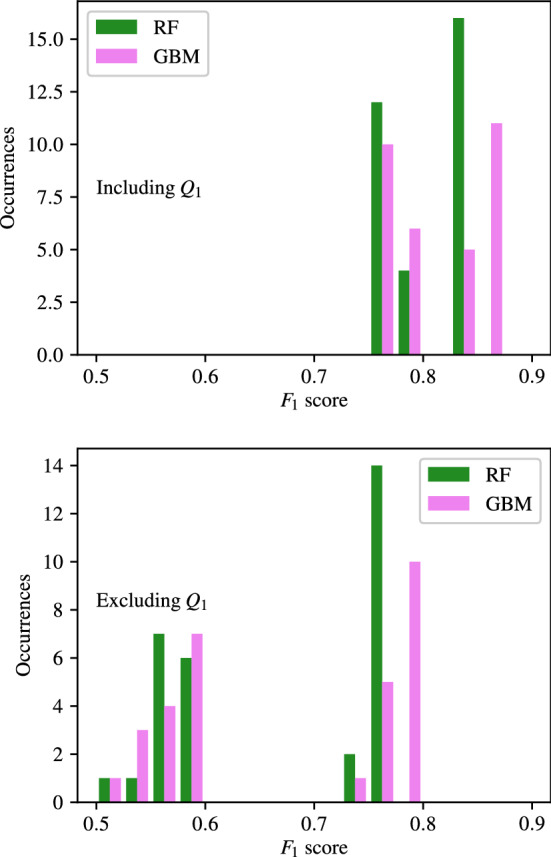
The histograms of the $F_1$ scores achieved for SAS classification are shown for all input measurement combinations that include $Q_1$ in the upper plot and exclude $Q_1$ in the lower plot

**Fig. 16:**
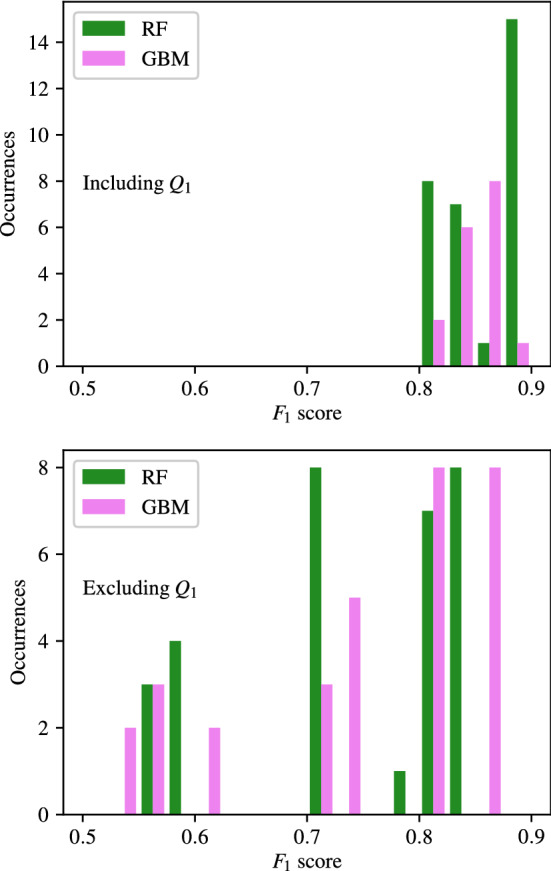
The histograms of the $F_1$ scores achieved for PAD classification are shown for all input measurement combinations that include $Q_1$ in the upper plot and exclude $Q_1$ in the lower plot

**Fig. 17:**
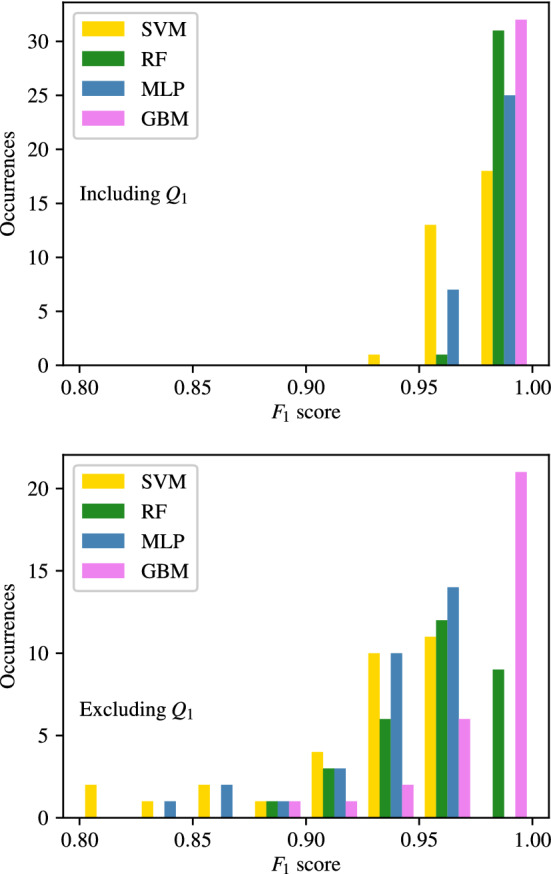
The histograms of the $F_1$ scores achieved for AAA classification are shown for all input measurement combinations that include $Q_1$ in the upper plot and exclude $Q_1$ in the lower plot

Appendices A–D, along with the above analysis show that classifiers trained using flow-rates in the common carotid arteries ($Q_1$) consistently produce the highest accuracy. To analyse this further, the $F_1$ scores of classifiers with combinations that include and exclude $Q_1$ are separated and compared for CAS, SAS, PAD, and AAA in Figs. 14, 15, 16, and 17, respectively. These figures show the the histograms of the $F_1$ scores, i.e. the number of occurrences/classifiers/combinations including and excluding $Q_1$ against $F_1$ score buckets. For each disease form, results are only shown for the classification methods that consistently produce good results for the corresponding disease form. The figures show a clear positive shift in the histograms when $Q_1$ is included, pointing to the particularly informative nature of $Q_1$. Other behaviours observed from these figures are:

- While there is generally an increase in $F_1$ score when including $Q_1$, it is also simultaneously observed that the maximum accuracies are relatively less sensitive to the inclusion of $Q_1$.
- The greatest distinction between $F_1$ scores when including or excluding $Q_1$ is seen for CAS classification when using the RF method. There is no overlap between the two RF histograms in Fig. 14.
- Observing the lower plots in Figs. 15 and 16, a clear subgroup of low-accuracy classifiers can be seen when excluding $Q_1$ for SAS and PAD, which does not exist when including $Q_1$.

### Feature importance

An important aspect of the GB method is that the measurement importance, which determines the influence that individual measurements have towards classification, can be computed. This split-improvement feature importance (Zhou and Hooker 2020) of a feature can be thought of as the contribution of that feature to the total information gain achieved in a decision tree, averaged across all the trees in the ensemble. A high feature importance suggests that the given feature is contributing heavily to the classification accuracies achieved. As the features provided to the GB classifiers are the FS coefficients describing the haemodynamic profiles, the total importance of each bilateral pressure or flow-rate measurement is found by summing the feature importance of the associated 22 FS coefficients. The total importance of each input measurement for each disease form is shown in Table 10.

**Table 10:** The total importance of each input measurement, based on the GB classifiers provided with all six measurements

	${Q}_1$ (%)	${Q}_2$ (%)	${Q}_3$ (%)	${P}_1$ (%)	${P}_2$ (%)	${P}_3$ (%)
*CAS*	67.38	8.02	3.89	11.07	7.692	1.93
*SAS*	41.90	29.98	8.40	6.80	5.97	6.921
*PAD*	38.01	15.98	31.11	4.62	4.63	5.62
*AAA*	69.34	19.10	4.95	2.41	2.61	1.55

Three important observations from this table are:

- The input measurement $Q_1$ consistently produces the highest importance for all forms of disease. This finding supports the findings of Sect. 3.2.
- The importance of each input measurement changes between disease forms based on the spatial proximity to the disease location. Generally, the measurements in close proximity to the disease location have higher importance. For example, the importance of $Q_3$ (flow-rate in the femoral arteries) is highest for PAD classification (see Fig. 1 for locations of disease and measurements). Similarly, $P_1$ (pressure in carotid arteries) has highest importance for CAS and SAS.
- The feature importances, when viewed in collection, also shed some light on why $Q_1$ is important for SAS and PAD even though the measurement location is far from the disease location. For SAS, the two most informative measurements are $Q_1$ and $Q_2$, and for PAD, these are $Q_1$ and $Q_3$. From Fig. 1, it is clear that these combinations form pairs of flow-rates before and after/at the disease location. Thus, the measurement locations bound the disease location to provide more information on the presence of disease.

### Lower severity aneurysms

In Sect. 3.1.4, it is found that AAAs can be classified to a very high levels of accuracy with only one input measurement. Whether these accuracies are affected when lower severity aneurysms are considered is assessed here. For this assessment, a new lower severity AAA VPD, referred to as $\text {VPD}_{\text {AAA-L}}$, is created in an identical manner to the other diseased databases (see Sect. 2.2), with the following two differences:

- The severity of aneurysms introduced into the virtual subjects (see Sect. 2.2.2) is sampled from a uniform distribution bounded as follows: $3.0 \le {\mathcal {S}}_{\text {aneurysm}} \le 7.0$.
- To reduce the computational expense associated with the creation of virtual patients, the size of $\text {VPD}_{\text {AAA-L}}$ is restricted to 5,000 VPs.

**Fig. 18:**
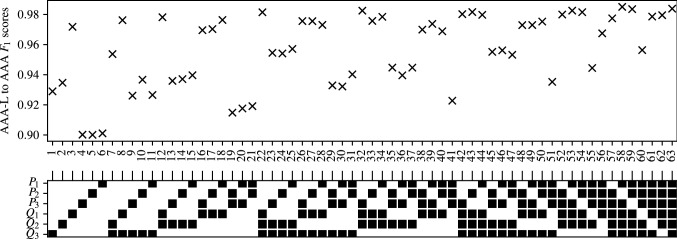
The ratios of the $F_1$ scores for AAA-L classification relative to AAA classification, when providing each combination of input measurements are shown. Measurements included within each combination are highlighted with a black square

A combination search is carried out with only the GB method as it is the best overall method. The $F_1$ scores, sensitivities, and specificities achieved by all the measurement combinations are presented in Appendix E. For comparison, the GB $F_1$ scores for all forms of disease (including AAA-L) are shown in Appendix F. The ratios of the GB $F_1$ scores achieved for AAA-L classification relative to AAA classification are shown in Fig. 18.

The observations from this figure are:

- The $F_1$ scores for AAA-L classification are consistently lower (ranging from 1% to 10% lower) than that for AAA classification. This finding supports the physiological expectation that the significance of biomarkers in pressure and flow-rate profiles is proportion to the severity.
- The ratios of $F_1$ scores are lowest for combinations of inputs that predominantly rely on pressure measurements. This suggests that pressure measurements are, in general, less informative about disease severity. This is in support of the, generally, lower feature importance of pressure measurements in Table 10.
- The $F_1$ score ratios are highest for input combinations that include $Q_1$. This finding further suggests that $Q_1$ contains consistent biomarkers.
- The ratios range between 0.9 and 0.99, implying a maximum degradation of only 10% relative to high-severity classification accuracies. Thus, even in the low-severity aneurysms, many combinations of classifiers achieve $F_1$ scores higher than 0.95 and corresponding sensitivities and specificities larger than 95%.

### Unilateral aneurysm measurement tests

Hitherto, all ML classifiers used bilateral measurements, *i.e.* both the right and left instances of each measurement were simultaneously provided. Here, the ability of unilateral measurements, *i.e.* only the right or left instance of a measurement, to detect AAAs is assessed. This analysis is restricted to the GB method as it consistently outperforms other methods.

GB classifiers are trained and tested to detect AAAs using four different unilateral measurements:

- *Flow-rate in the right carotid artery*, shown in Fig. 1 as $Q_1^{\text {(R)}}$.
- *Flow-rate in the left carotid artery*, shown in Fig. 1 as $Q_1^{\text {(L)}}$.
- *Pressure in the right radial artery*, shown in Fig. 1 as $P_3^{\text {(R)}}$.
- *Pressure in the left radial artery*, shown in Fig. 1 as $P_3^{\text {(L)}}$.

Carotid artery flow-rate is chosen as it has been shown to be the best measurement for disease classification. Radial artery pressure is chosen due to the location of the radial artery on the human wrist. Recent advancements have resulted in wearable devices capable of measuring continuous radial pressure profiles, such as the TLT Sapphire monitor (Tarilian Laser Technologies, Welwyn Garden City, U.K.) (Lobo et al. 2019), and thus if AAAs can be detected to satisfactory accuracies using these measurements, it may suggest the possibility of future home monitoring of abdominal aortic health through such wearables. The sensitivities and specificities achieved by the four unilateral GB classifiers are shown in Table 11. It shows that relative to the bilateral case, while there is a decrease in the classification accuracies, the magnitude of the decrease is less than 10%. This finding suggests that there may be sufficient biomarkers of AAA presence captured within the intra-measurement details of a single pressure or flow-rate profile. The fact that similar accuracies are achieved with either the right or left instances of any measurement is likely due to physiological symmetry. While there are some minor asymmetries between the right and left upper extremities, due to the topology of the arterial network (as shown in Fig. 1) changes to the cross-sectional area of the abdominal aorta are expected to produce relatively consistent changes in both the right and left side of the body.

**Table 11:** The sensitivities and specificities achieved when using the measurements of flow-rate in the right, left, and both CAs and pressure in the right, left, and both radial arteries

	Side	Sensitivity	Specificity
Carotid	Right	0.9369	0.9161
Flow-rate	Left	0.9065	0.9146
($Q_1$)	Both	0.9799	0.9811
Radial	Right	0.8356	0.8533
Pressure	Left	0.8633	0.8605
($P_3$)	Both	0.9202	0.9248

### MLP early stopping to avoid overfitting

It is shown in Sect. 3.1.1 that the accuracy of MLP classifiers is hindered by the presence of overfitting. Thus, the early stopping criterion outlined in Sect. 2.7 is adopted for the combinations of three to six measurements that hitherto produced best results without early stopping. Here, the hyper-parameters describing the MLP architecture—the number of neurons per layer and the number of layers (depth)—for each such case are also individually re-optimised on the validation data set with early stopping criterion enabled. Thus, for each combination in the grid search, the best validation set $F_1$ score is computed with early stopping enabled during training, and the architecture producing the maximum $F_1$ score is selected. Subsequently, for this optimal architecture, the test scores are computed on the test data set. This analysis is performed for CAS and AAA as the behaviour of SAS and PAD is very similar to that of CAS.

**Fig. 19:**
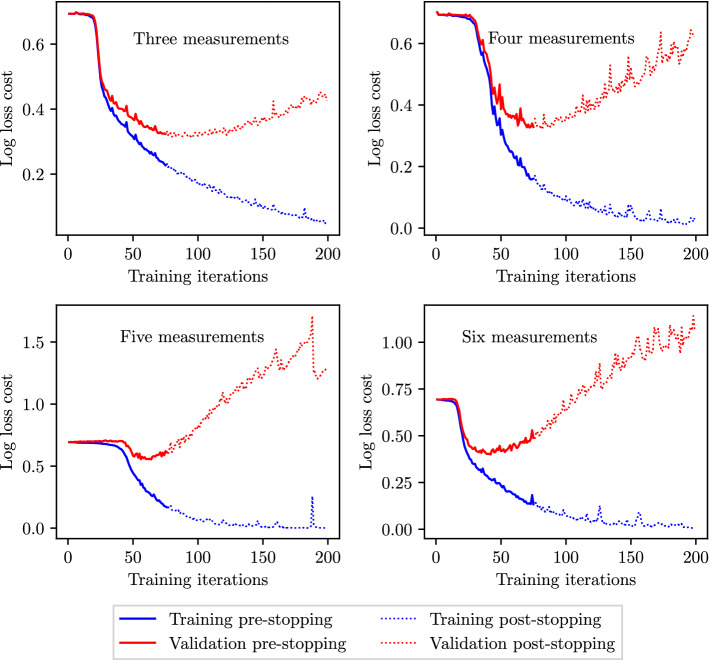
MLP: the log loss cost profiles across the training and validation sets when using the best performing combination containing three to six input measurements for CAS classification and employing early stopping

#### CAS: early stopping

The hyper-parameters describing the optimum architectures with early stopping criterion for best combinations are shown in Table 12. It shows a remarkable degree of consistency between the optimum hyper-parameters for varying number of input measurements: for four measurements and above the optimal architecture is identical. This finding supports the previous simplification of using a single architecture for all the MLP classifiers. An interesting finding to note, however, is that there is less consistency with the previous optimum hyper-parameters presented in Table 4, which found that four layers containing 60 neurons produced the highest $F_1$ score when providing six input measurements.

The cost profiles for the optimal architectures with early stopping are shown in Fig. 19. It shows that generally the early stopping criteria fulfil its purpose of stopping the training process near to the minimum validation cost point, thus minimising overfitting. It is observed that for all numbers of input measurements, training is stopped as soon as the 75 minimum iterations have been completed. While this early stopping criteria greatly reduce overfitting in all the cases, it is seen that the minimum number of training iterations (75) is too high for the six measurement case (the validation cost has already started to significantly rise), suggesting further refinement may reduce the validation and test costs even further.

A comparison between the $F_1$ scores achieved with and without early stopping is shown in Table 13. While early stopping has reduced the log loss cost across the validation and test sets, this does not necessarily translate to improvements in the $F_1$ score. The log loss cost will decrease without increasing the $F_1$ score if easy to classify patients are predicted with a higher degree of certainty (for example, predicting 95% probability rather than 75%) even if no new additional patients are correctly classified. For the six-measurement case, however, some increase in $F_1$ score is clearly observed as a benefit of early stopping.

**Table 12:** The hyper-parameters describing the architecture of the MLP classifiers that produce the highest $F_1$ scores on the validation set with early stopping criterion for CAS classification, when using the best performing combinations of three to six input measurements

No. measurements & combination	Neurons per layer	# of layers	${F}_1$ (validation)
3 – ($P_3$, $P_2$, $P_1$)	140	3	0.8817
4 – ($Q_3$, $Q_1$, $P_2$, $P_1$)	180	4	0.8824
5 – ($Q_3$, $Q_2$, $P_3$, $P_2$, $P_1$)	180	4	0.8355
6 – ($Q_3$, $Q_2$, $Q_1$, $P_3$, $P_2$, $P_1$)	180	4	0.8464

**Table 13:** MLP: $F_1$ scores on the test dataset when using the best three to six input measurement combinations found to produce the highest accuracies for CAS with (Sect. 3.6.1) and without early stopping (Sect. 3.1.1)

Number of input measurements	Combination	$F_1$ score (test)	
		Without early stopping	With early stopping
3	($P_3$, $P_2$, $P_1$)	0.8831	0.8621
4	($Q_3$, $Q_1$, $P_2$, $P_1$)	0.8683	0.8693
5	($Q_3$, $Q_2$, $P_3$, $P_2$, $P_1$)	0.8463	0.7975
6	($Q_3$, $Q_2$, $Q_1$, $P_3$, $P_2$, $P_1$)	0.7785	0.8394

#### AAA: early stopping

The hyper-parameters describing theoptimum architectures with early stopping criterion for best combinations are shown in Table 14. The consistency of best architecture for AAA across the number of measurements is less when compared to that for CAS. It is again observed that the new hyper-parameters are inconsistent with the old (Table 4). Initially, this finding may seem to undermine early stopping and individual architecture optimisation for varying number of input measurements. However, while the optimum hyper-parameters are inconsistent, the $F_1$ scores achieved are very similar—0.9785 in Table 4 and 0.9870 in Table 14. This similarity in $F_1$ scores may suggest an insusceptibility to the architecture used, *i.e.* the $F_1$ score plane in the two-dimensional grid-search space is relatively flat for this problem. This again supports the earlier simplification of using a single architecture for all the classifiers.

**Fig. 20:**
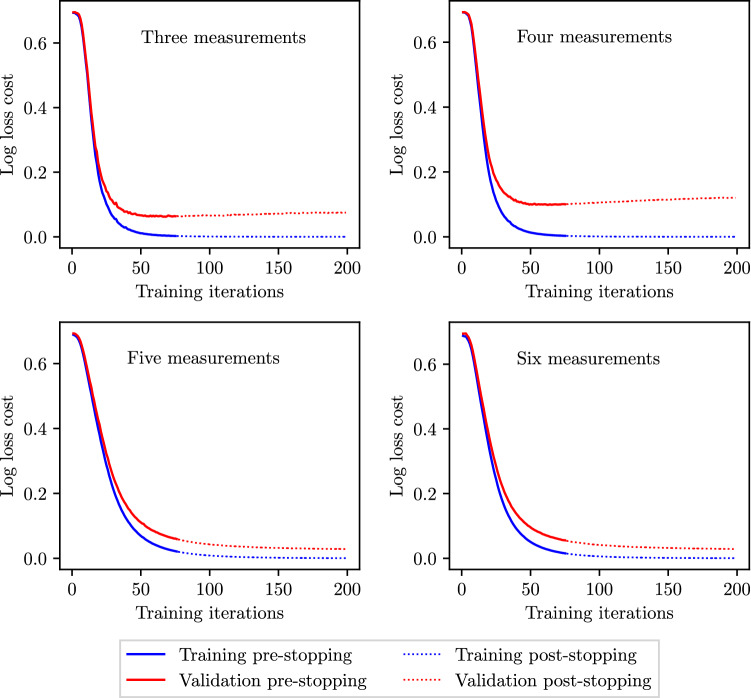
MLP: the log loss cost profiles across the training and validation sets when using the best performing combination containing three to six input measurements for AAA classification and employing early stopping

The cost profiles for the optimal architectures with early stopping are shown in Fig. 20. It shows no major signs of overfitting when using MLP classifiers to detect AAA. As a result, the employment of an early stopping criteria has little affect on the final log loss cost achieved across all training and validation data sets. Thus, when comparing the with and without early stopping test scores in Table 15, no significant differences in the $F_1$ scores achieved are observed for AAA classification.

The aforementioned findings with early stopping enabled for both CAS and AAA classification, suggest that to substantially improve the accuracy of MLP classifiers, a more extensive hyper-parameter optimisation strategy, which tunes many other hyper-parameters, is required, and should be adopted in future studies.

**Table 14:** The hyper-parameters describing the architecture of the MLP classifiers that produce the highest $F_1$ scores on the validation set with early stopping criterion for AAA classification, when using the best performing combinations of three to six input measurements

No. measurements & combination	Neurons per layer	# of layers	${F}_1$ (validation)
3 – ($Q_1$, $P_2$, $P_1$)	140	2	0.9889
4 – ($Q_2$, $Q_1$, $P_2$, $P_1$)	60	2	0.9858
5 – ($Q_2$, $Q_1$, $P_3$, $P_2$, $P_1$)	150	1	0.9915
6 – ($Q_3$, $Q_2$, $Q_1$, $P_3$, $P_2$, $P_1$)	160	1	0.9870

**Table 15:** MLP: $F_1$ scores on the test dataset when using the best three to six input measurement combinations found to produce the highest accuracies for AAA with (Sect. 3.6.2) and without early stopping (Sect. 3.1.4)

Number of input measurements	Combination	$F_1$ score	
		Without early stopping	With early stopping
3	($Q_1$, $P_2$, $P_1$)	0.9827	0.9852
4	($Q_2$, $Q_1$, $P_2$, $P_1$)	0.9800	0.9784
5	($Q_2$, $Q_1$, $P_3$, $P_2$, $P_1$)	0.9808	0.9876
6	($Q_3$, $Q_2$, $Q_1$, $P_3$, $P_2$, $P_1$)	0.9785	0.9836

## Conclusions

The main conclusion of this study is that machine learning methods have the potential to detect arterial disease—both stenoses and aneurysms—from peripheral measurements of pressure and flow-rates across the network. Amongst various ML methods, it is found that tree-based methods of Random Forest and Gradient Boosting perform best for this application (within the limitations of the classifier specific optimisation performed). Across the different forms of disease, the Gradient Boosting method outperforms Random Forest, Support Vector Machine, Naive Bayes, Logistic Regression, and even the deep learning method of Multi-layer Perceptron in the setting adopted. It should be noted, however, that the multi-layer perceptron results could be improved by problem specific optimisation of architecture and fine-tuning of further hyper-parameters. This, however, would come at added complexity and computational costs against the easier-to-train machine-learning methods of Random Forest and Gradient Boosting.

It is demonstrated that maximum $F_1$ scores larger than 0.9 are achievable for CAS and PAD, larger than 0.85 for SAS, and larger than 0.98 for both low- and high-severity AAAs. The corresponding sensitivities and specificities are also both larger than 90% for CAS and PAD, larger than 85% for SAS, and larger than 98% for both low- and high-severity AAAs. While these maximum scores are for the case when all the six measurements are used, it is also shown that the performance degradation is less than 5% when using only three measurements and less than 10% when using only two measurements, as long as the these measurements are carefully chosen in specific combinations. For the case of AAA, it is further demonstrated that when only a single measurement (either on the left or right side) is used, $F_1$ scores larger than 0.85 and corresponding sensitivities and specificities larger than 85% are achievable. This aspect encourages the application of AAA monitoring and/or screening through the use of a wearable device, such at the TLT Sapphire monitor (Tarilian Laser Technologies, Welwyn Garden City, U.K.) (Lobo et al. 2019). Confidence in this is further strengthened by the similar very high accuracies reported for AAA classification by Chakshu et al. (2020) ($\approx 99.9\%$) and Wang et al. (2021) (sensitivities and specificities of $\approx 86\%$). However, multi-class classifier accuracies, as opposed to only the binary classifiers assessed here, remain unknown and should be considered to fully assess the ability of machine and deep learning methods for arterial disease detection.

Finally, it is shown through the analysis of several classifiers and feature-importance that, among the measurements, the carotid artery flow-rate is a particularly informative measurement to detect the presence of all the four forms of disease considered.

## Limitations & future work

While high accuracy classification has been achieved, all classifiers are binary (*i.e.* disease are treated mutually exclusively). A logical next step, to further the results presented here, is to relax the assumption of mutually exclusive disease. Thus, classifiers should be built to detect not only the presence of disease, but also identify the type of disease (potentially concomitant disease in multiple locations), its location, and its severity. This further analysis can be completed in two stages:

1. The previously created unhealthy VPDs (each containing only one form of disease) can be used to created mixed disease data sets, *i.e.* each VP has only one form of disease; however, the data sets contain multiple forms of disease. Binary ML classifiers can then be created to predict if a VP is subject to a particular form of disease, and multiclass classifiers to determine which form of disease a VP has.
2. New VPDs can be created, in which each VP may contain more than one form of disease. In this case, binary classifiers can be created to predict the presence of each individual form of disease within a VP, and multiclass classifiers to predict the combination of disease forms present.

While the results are encouraging, they are produced on a virtual cohort of subjects. Even though the database is physiologically realistic and carefully constructed, it may be that real patient behaviour differs from those in the VPD. Therefore, future steps should be in applying the trained classifiers here directly to a small cohort of real-patient measurements. The effect of measurement errors and biases is ignored in this study. This aspect can also be considered in future studies. Further improvements can be also made, to aim for higher accuracies with fewer, potentially noise- and bias-corrupted, measurements, by:

- Further optimising the architectures of the machine and deep learning methods (particularly MLP classifiers).
- Further monitoring individual classifiers for signs of overfitting, and minimising this when needed.

## Figures and Tables

**Fig. 21:**
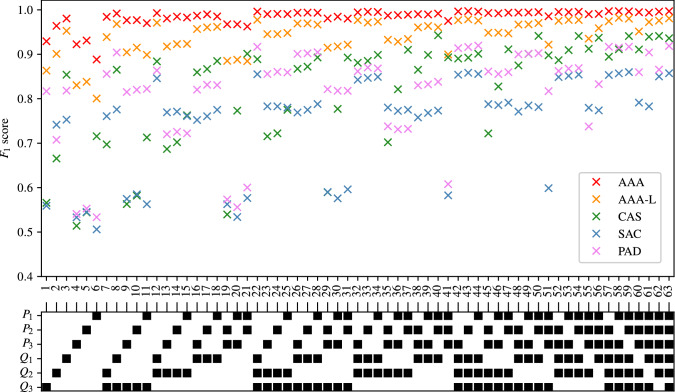
The $F_1$ scores achieved for all disease forms when employing the GB method. Measurements included within each combination are highlighted with a black square

## CAS combination search results

The $F_1$ scores, sensitivities, and specificities achieved for CAS classification when using each of the six ML methods are shown in Tables 16, 17, and 18, respectively.

**Table 16:** The $F_1$ scores achieved across the combination search by each of the six classification methods

Input combination	Classification method					
	NB	LR	SVM	RF	MLP	GB
$Q_3$	0.5547	0.5110	0.5157	0.5807	0.4365	0.5606
$Q_2$	0.5105	0.5080	0.4955	0.6858	0.4410	0.6565
$Q_1$	0.5676	0.5033	0.5953	0.8809	0.6459	0.8521
$P_3$	0.4927	0.5023	0.4991	0.5441	0.4805	0.5131
$P_2$	0.4413	0.5066	0.5260	0.5628	0.3741	0.5412
$P_1$	0.5473	0.4917	0.5712	0.6681	0.7013	0.7082
$Q_3$, $Q_2$	0.5684	0.4955	0.5104	0.6955	0.4915	0.6889
$Q_3$, $Q_1$	0.4831	0.5050	0.5544	0.8790	0.6944	0.8629
$Q_3$, $P_3$	0.5213	0.4935	0.5124	0.5825	0.4929	0.5659
$Q_3$, $P_2$	0.5853	0.5018	0.5142	0.5918	0.4904	0.5849
$Q_3$, $P_1$	0.5048	0.5034	0.5576	0.6601	0.6864	0.7105
$Q_2$, $Q_1$	0.4600	0.4975	0.5540	0.8913	0.6648	0.8824
$Q_2$, $P_3$	0.4804	0.4940	0.5109	0.6833	0.4158	0.6805
$Q_2$, $P_2$	0.5290	0.5037	0.5125	0.6836	0.5618	0.6908
$Q_2$, $P_1$	0.4434	0.4978	0.5597	0.7204	0.6741	0.7562
$Q_1$, $P_3$	0.4470	0.4990	0.5595	0.8732	0.6860	0.8577
$Q_1$, $P_2$	0.5341	0.5029	0.5629	0.8774	0.7090	0.8684
$Q_1$, $P_1$	0.4927	0.5018	0.6233	0.8837	0.7822	0.8850
$P_3$, $P_2$	0.5507	0.5117	0.5263	0.5581	0.5313	0.5431
$P_3$, $P_1$	0.5266	0.4963	0.5725	0.6837	0.7384	0.7539
$P_2$, $P_1$	0.5089	0.4944	0.6885	0.7938	0.8878	0.8950
$Q_3$, $Q_2$, $Q_1$	0.4299	0.4995	0.5425	0.8907	0.6838	0.8868
$Q_3$, $Q_2$, $P_3$	0.4822	0.4980	0.5058	0.6910	0.5300	0.7072
$Q_3$, $Q_2$, $P_2$	0.5346	0.4975	0.5204	0.6962	0.5211	0.7102
$Q_3$, $Q_2$, $P_1$	0.5267	0.5024	0.5428	0.7229	0.6084	0.7693
$Q_3$, $Q_1$, $P_3$	0.4636	0.5016	0.5317	0.8685	0.6699	0.8660
$Q_3$, $Q_1$, $P_2$	0.5186	0.4960	0.5580	0.8751	0.6469	0.8728
$Q_3$, $Q_1$, $P_1$	0.5257	0.5020	0.5888	0.8843	0.7532	0.8903
$Q_3$, $P_3$, $P_2$	0.4493	0.5032	0.5119	0.5923	0.5418	0.5888
$Q_3$, $P_3$, $P_1$	0.5019	0.4892	0.5527	0.6751	0.7159	0.7602
$Q_3$, $P_2$, $P_1$	0.4312	0.5042	0.6303	0.7564	0.8623	0.8923
$Q_2$, $Q_1$, $P_3$	0.5222	0.5041	0.5300	0.8840	0.6354	0.8776
$Q_2$, $Q_1$, $P_2$	0.5155	0.4957	0.5586	0.8847	0.7001	0.8844
$Q_2$, $Q_1$, $P_1$	0.5251	0.4940	0.6039	0.8941	0.7611	0.8968
$Q_2$, $P_3$, $P_2$	0.4893	0.5041	0.5241	0.6824	0.5335	0.6929
$Q_2$, $P_3$, $P_1$	0.4067	0.4965	0.5421	0.7249	0.7185	0.8064
$Q_2$, $P_2$, $P_1$	0.5479	0.4858	0.6415	0.7740	0.8735	0.9040
$Q_1$, $P_3$, $P_2$	0.4766	0.4969	0.5505	0.8700	0.7048	0.8651
$Q_1$, $P_3$, $P_1$	0.5037	0.4908	0.5975	0.8777	0.7645	0.8956
$Q_1$, $P_2$, $P_1$	0.4997	0.4972	0.6772	0.8872	0.8680	0.9389
$P_3$, $P_2$, $P_1$	0.5090	0.4997	0.6451	0.7694	0.8831	0.8936
$Q_3$, $Q_2$, $Q_1$, $P_3$	0.4569	0.4921	0.5408	0.8835	0.6258	0.8855
$Q_3$, $Q_2$, $Q_1$, $P_2$	0.4253	0.5022	0.5462	0.8871	0.6655	0.8887
$Q_3$, $Q_2$, $Q_1$, $P_1$	0.4934	0.5068	0.5783	0.8925	0.7163	0.9004
$Q_3$, $Q_2$, $P_3$, $P_2$	0.4875	0.5026	0.5234	0.6852	0.5483	0.7145
$Q_3$, $Q_2$, $P_3$, $P_1$	0.4481	0.4945	0.5399	0.7231	0.6714	0.8125
$Q_3$, $Q_2$, $P_2$, $P_1$	0.4329	0.5034	0.6043	0.7619	0.8618	0.9025
$Q_3$, $Q_1$, $P_3$, $P_2$	0.4934	0.4972	0.5400	0.8717	0.6299	0.8761
$Q_3$, $Q_1$, $P_3$, $P_1$	0.5365	0.5011	0.5802	0.8789	0.7197	0.8978
$Q_3$, $Q_1$, $P_2$, $P_1$	0.5068	0.4974	0.6338	0.8852	0.8542	0.9395
$Q_3$, $P_3$, $P_2$, $P_1$	0.4329	0.4980	0.6137	0.7393	0.8471	0.8906
$Q_2$, $Q_1$, $P_3$, $P_2$	0.5669	0.4933	0.5468	0.8822	0.6524	0.8844
$Q_2$, $Q_1$, $P_3$, $P_1$	0.5193	0.4978	0.5783	0.8878	0.7207	0.9065
$Q_2$, $Q_1$, $P_2$, $P_1$	0.4638	0.5037	0.6413	0.8944	0.8683	0.9383
$Q_2$, $P_3$, $P_2$, $P_1$	0.4868	0.4999	0.6142	0.7694	0.8503	0.9084
$Q_1$, $P_3$, $P_2$, $P_1$	0.4735	0.5025	0.6320	0.8807	0.8547	0.9353
$Q_3$, $Q_2$, $Q_1$, $P_3$, $P_2$	0.5005	0.5015	0.5387	0.8848	0.6322	0.8927
$Q_3$, $Q_2$, $Q_1$, $P_3$, $P_1$	0.4652	0.4962	0.5760	0.8875	0.7079	0.9093
$Q_3$, $Q_2$, $Q_1$, $P_2$, $P_1$	0.5108	0.4994	0.6088	0.8934	0.8313	0.9381
$Q_3$, $Q_2$, $P_3$, $P_2$, $P_1$	0.4994	0.5105	0.5808	0.7540	0.8463	0.9052
$Q_3$, $Q_1$, $P_3$, $P_2$, $P_1$	0.5330	0.5024	0.6108	0.8849	0.8380	0.9364
$Q_2$, $Q_1$, $P_3$, $P_2$, $P_1$	0.4899	0.5054	0.6026	0.8900	0.8371	0.9391
$Q_3$, $Q_2$, $Q_1$, $P_3$, $P_2$, $P_1$	0.4634	0.5018	0.5862	0.8878	0.7785	0.9343

**Table 17:** The sensitivities achieved across the combination search by each of the six classification methods

Input combination	Classification method					
	NB	LR	SVM	RF	MLP	GB
$Q_3$	0.1531	0.5527	0.4283	0.5572	0.6084	0.5736
$Q_2$	0.6641	0.5097	0.6418	0.6575	0.6228	0.6744
$Q_1$	0.5426	0.4525	0.5694	0.8704	0.4243	0.8547
$P_3$	0.5024	0.5098	0.4999	0.5139	0.5355	0.5158
$P_2$	0.6490	0.4979	0.5052	0.5366	0.7038	0.5491
$P_1$	0.2410	0.4992	0.6588	0.6510	0.7052	0.7215
$Q_3$, $Q_2$	0.5055	0.5035	0.5651	0.6655	0.5439	0.7044
$Q_3$, $Q_1$	0.7217	0.5094	0.5461	0.8681	0.6288	0.8661
$Q_3$, $P_3$	0.4462	0.5091	0.4944	0.5615	0.5226	0.5583
$Q_3$, $P_2$	0.3652	0.5090	0.5111	0.5731	0.5572	0.5777
$Q_3$, $P_1$	0.3032	0.5049	0.6510	0.6411	0.7334	0.7149
$Q_2$, $Q_1$	0.4543	0.5091	0.5770	0.8765	0.6372	0.8845
$Q_2$, $P_3$	0.5395	0.5117	0.5263	0.6555	0.6691	0.6926
$Q_2$, $P_2$	0.6043	0.4987	0.5436	0.6563	0.3867	0.7117
$Q_2$, $P_1$	0.5766	0.4905	0.6442	0.6962	0.6879	0.7668
$Q_1$, $P_3$	0.4718	0.5003	0.5461	0.8679	0.7167	0.8601
$Q_1$, $P_2$	0.3903	0.5068	0.5438	0.8672	0.7467	0.8664
$Q_1$, $P_1$	0.6488	0.4978	0.6672	0.8798	0.8290	0.8848
$P_3$, $P_2$	0.4744	0.5034	0.5251	0.5383	0.4863	0.5335
$P_3$, $P_1$	0.3334	0.4815	0.6303	0.6599	0.7469	0.7842
$P_2$, $P_1$	0.4943	0.5001	0.6699	0.7762	0.8995	0.9026
$Q_3$, $Q_2$, $Q_1$	0.4935	0.4812	0.5598	0.8789	0.6425	0.8896
$Q_3$, $Q_2$, $P_3$	0.5586	0.4913	0.5454	0.6647	0.5014	0.7219
$Q_3$, $Q_2$, $P_2$	0.4348	0.5091	0.5336	0.6788	0.5298	0.7313
$Q_3$, $Q_2$, $P_1$	0.2964	0.5165	0.6068	0.6975	0.6471	0.7783
$Q_3$, $Q_1$, $P_3$	0.4427	0.5021	0.5321	0.8620	0.6388	0.8677
$Q_3$, $Q_1$, $P_2$	0.4268	0.4974	0.5605	0.8718	0.6645	0.8721
$Q_3$, $Q_1$, $P_1$	0.5134	0.4687	0.6341	0.8735	0.7474	0.8936
$Q_3$, $P_3$, $P_2$	0.6823	0.4939	0.5076	0.5698	0.4853	0.5905
$Q_3$, $P_3$, $P_1$	0.3887	0.4941	0.5882	0.6691	0.6907	0.7866
$Q_3$, $P_2$, $P_1$	0.6288	0.4919	0.6251	0.7473	0.8593	0.8928
$Q_2$, $Q_1$, $P_3$	0.4599	0.5056	0.5474	0.8735	0.6999	0.8819
$Q_2$, $Q_1$, $P_2$	0.4296	0.5085	0.5728	0.8766	0.6703	0.8853
$Q_2$, $Q_1$, $P_1$	0.5235	0.5037	0.6393	0.8825	0.7635	0.8991
$Q_2$, $P_3$, $P_2$	0.3581	0.4902	0.5484	0.6566	0.5249	0.7098
$Q_2$, $P_3$, $P_1$	0.6042	0.4816	0.6079	0.7001	0.7140	0.8277
$Q_2$, $P_2$, $P_1$	0.5758	0.4826	0.6481	0.7516	0.8780	0.9114
$Q_1$, $P_3$, $P_2$	0.4530	0.4984	0.5521	0.8616	0.6733	0.8651
$Q_1$, $P_3$, $P_1$	0.4760	0.4859	0.6233	0.8721	0.7403	0.8993
$Q_1$, $P_2$, $P_1$	0.4610	0.4917	0.6744	0.8807	0.8797	0.9433
$P_3$, $P_2$, $P_1$	0.4001	0.5159	0.6442	0.7562	0.8731	0.8930
$Q_3$, $Q_2$, $Q_1$, $P_3$	0.5792	0.4903	0.5548	0.8700	0.6711	0.8916
$Q_3$, $Q_2$, $Q_1$, $P_2$	0.6211	0.4961	0.5726	0.8788	0.6622	0.8933
$Q_3$, $Q_2$, $Q_1$, $P_1$	0.5018	0.4831	0.5948	0.8837	0.7442	0.9015
$Q_3$, $Q_2$, $P_3$, $P_2$	0.5499	0.4981	0.4938	0.6722	0.4844	0.7277
$Q_3$, $Q_2$, $P_3$, $P_1$	0.4381	0.5052	0.5948	0.7055	0.6997	0.8337
$Q_3$, $Q_2$, $P_2$, $P_1$	0.7010	0.5049	0.6401	0.7463	0.8600	0.9129
$Q_3$, $Q_1$, $P_3$, $P_2$	0.4629	0.5000	0.5424	0.8597	0.6331	0.8747
$Q_3$, $Q_1$, $P_3$, $P_1$	0.4436	0.4937	0.6099	0.8747	0.7555	0.9017
$Q_3$, $Q_1$, $P_2$, $P_1$	0.5362	0.5062	0.6300	0.8761	0.8538	0.9417
$Q_3$, $P_3$, $P_2$, $P_1$	0.6073	0.5011	0.6165	0.7243	0.8391	0.8993
$Q_2$, $Q_1$, $P_3$, $P_2$	0.4973	0.5056	0.5779	0.8729	0.6427	0.8874
$Q_2$, $Q_1$, $P_3$, $P_1$	0.4225	0.5065	0.6115	0.8813	0.7596	0.9100
$Q_2$, $Q_1$, $P_2$, $P_1$	0.5115	0.4954	0.6345	0.8858	0.8618	0.9416
$Q_2$, $P_3$, $P_2$, $P_1$	0.5582	0.4877	0.6266	0.7498	0.8573	0.9133
$Q_1$, $P_3$, $P_2$, $P_1$	0.5769	0.4891	0.6309	0.8674	0.8667	0.9375
$Q_3$, $Q_2$, $Q_1$, $P_3$, $P_2$	0.5446	0.4929	0.5686	0.8759	0.6487	0.8949
$Q_3$, $Q_2$, $Q_1$, $P_3$, $P_1$	0.4676	0.4933	0.6021	0.8775	0.7169	0.9117
$Q_3$, $Q_2$, $Q_1$, $P_2$, $P_1$	0.5403	0.5015	0.6142	0.8858	0.8288	0.9415
$Q_3$, $Q_2$, $P_3$, $P_2$, $P_1$	0.6396	0.5042	0.6070	0.7375	0.8308	0.9120
$Q_3$, $Q_1$, $P_3$, $P_2$, $P_1$	0.5330	0.4920	0.6171	0.8795	0.8345	0.9399
$Q_2$, $Q_1$, $P_3$, $P_2$, $P_1$	0.4640	0.4919	0.6149	0.8774	0.8273	0.9416
$Q_3$, $Q_2$, $Q_1$, $P_3$, $P_2$, $P_1$	0.6224	0.5116	0.6012	0.8747	0.7916	0.9364

**Table 18:** The specificities achieved across the combination search by each of the six classification methods

Input combination	Classification method					
	NB	LR	SVM	RF	MLP	GB
$Q_3$	0.7090	0.4968	0.5462	0.5904	0.3886	0.5556
$Q_2$	0.4579	0.5075	0.4474	0.7006	0.3896	0.6479
$Q_1$	0.5776	0.5204	0.6063	0.8893	0.7517	0.8502
$P_3$	0.4896	0.4998	0.4989	0.5555	0.4632	0.5122
$P_2$	0.3826	0.5096	0.5335	0.5732	0.2983	0.5384
$P_1$	0.6628	0.4893	0.5363	0.6767	0.6993	0.7010
$Q_3$, $Q_2$	0.5935	0.4929	0.4917	0.7116	0.4745	0.6808
$Q_3$, $Q_1$	0.4072	0.5036	0.5576	0.8876	0.7293	0.8605
$Q_3$, $P_3$	0.5478	0.4884	0.5187	0.5912	0.4832	0.5690
$Q_3$, $P_2$	0.6764	0.4995	0.5153	0.5998	0.4687	0.5879
$Q_3$, $P_1$	0.5730	0.5030	0.5215	0.6696	0.6619	0.7081
$Q_2$, $Q_1$	0.4618	0.4937	0.5453	0.9032	0.6786	0.8808
$Q_2$, $P_3$	0.4618	0.4883	0.5057	0.6978	0.3494	0.6743
$Q_2$, $P_2$	0.5020	0.5055	0.5018	0.6978	0.6303	0.6798
$Q_2$, $P_1$	0.4055	0.5003	0.5269	0.7341	0.6672	0.7498
$Q_1$, $P_3$	0.4399	0.4987	0.5648	0.8774	0.6701	0.8560
$Q_1$, $P_2$	0.5865	0.5016	0.5704	0.8855	0.6883	0.8701
$Q_1$, $P_1$	0.4417	0.5032	0.6035	0.8869	0.7522	0.8853
$P_3$, $P_2$	0.5798	0.5146	0.5268	0.5659	0.5477	0.5467
$P_3$, $P_1$	0.5958	0.5013	0.5494	0.6961	0.7335	0.7356
$P_2$, $P_1$	0.5140	0.4926	0.6983	0.8054	0.8785	0.8889
$Q_3$, $Q_2$, $Q_1$	0.4125	0.5057	0.5361	0.9002	0.7054	0.8846
$Q_3$, $Q_2$, $P_3$	0.4580	0.5003	0.4925	0.7049	0.5404	0.6992
$Q_3$, $Q_2$, $P_2$	0.5711	0.4937	0.5158	0.7055	0.5181	0.6986
$Q_3$, $Q_2$, $P_1$	0.6091	0.4977	0.5190	0.7374	0.5915	0.7638
$Q_3$, $Q_1$, $P_3$	0.4700	0.5015	0.5316	0.8735	0.6857	0.8648
$Q_3$, $Q_1$, $P_2$	0.5508	0.4956	0.5571	0.8777	0.6386	0.8734
$Q_3$, $Q_1$, $P_1$	0.5302	0.5132	0.5699	0.8929	0.7568	0.8877
$Q_3$, $P_3$, $P_2$	0.3818	0.5064	0.5135	0.6018	0.5629	0.5882
$Q_3$, $P_3$, $P_1$	0.5399	0.4877	0.5392	0.6782	0.7300	0.7441
$Q_3$, $P_2$, $P_1$	0.3769	0.5084	0.6328	0.7620	0.8646	0.8920
$Q_2$, $Q_1$, $P_3$	0.5443	0.5037	0.5238	0.8924	0.6055	0.8743
$Q_2$, $Q_1$, $P_2$	0.5454	0.4916	0.5531	0.8913	0.7162	0.8838
$Q_2$, $Q_1$, $P_1$	0.5258	0.4909	0.5886	0.9035	0.7597	0.8950
$Q_2$, $P_3$, $P_2$	0.5319	0.5088	0.5156	0.6959	0.5367	0.6840
$Q_2$, $P_3$, $P_1$	0.3563	0.5015	0.5177	0.7390	0.7211	0.7921
$Q_2$, $P_2$, $P_1$	0.5374	0.4869	0.6385	0.7882	0.8701	0.8979
$Q_1$, $P_3$, $P_2$	0.4840	0.4965	0.5500	0.8766	0.7220	0.8651
$Q_1$, $P_3$, $P_1$	0.5131	0.4924	0.5866	0.8822	0.7795	0.8927
$Q_1$, $P_2$, $P_1$	0.5126	0.4991	0.6787	0.8925	0.8592	0.9351
$P_3$, $P_2$, $P_1$	0.5462	0.4944	0.6456	0.7777	0.8911	0.8942
$Q_3$, $Q_2$, $Q_1$, $P_3$	0.4208	0.4928	0.5357	0.8943	0.6053	0.8807
$Q_3$, $Q_2$, $Q_1$, $P_2$	0.3725	0.5043	0.5363	0.8938	0.6672	0.8852
$Q_3$, $Q_2$, $Q_1$, $P_1$	0.4907	0.5149	0.5716	0.8996	0.7008	0.8995
$Q_3$, $Q_2$, $P_3$, $P_2$	0.4675	0.5042	0.5340	0.6921	0.5725	0.7072
$Q_3$, $Q_2$, $P_3$, $P_1$	0.4510	0.4911	0.5196	0.7331	0.6572	0.7981
$Q_3$, $Q_2$, $P_2$, $P_1$	0.3589	0.5029	0.5888	0.7716	0.8632	0.8941
$Q_3$, $Q_1$, $P_3$, $P_2$	0.5034	0.4963	0.5392	0.8811	0.6285	0.8773
$Q_3$, $Q_1$, $P_3$, $P_1$	0.5706	0.5037	0.5681	0.8823	0.6997	0.8947
$Q_3$, $Q_1$, $P_2$, $P_1$	0.4969	0.4946	0.6357	0.8926	0.8545	0.9376
$Q_3$, $P_3$, $P_2$, $P_1$	0.3848	0.4970	0.6126	0.7481	0.8530	0.8837
$Q_2$, $Q_1$, $P_3$, $P_2$	0.5945	0.4893	0.5352	0.8896	0.6571	0.8822
$Q_2$, $Q_1$, $P_3$, $P_1$	0.5533	0.4950	0.5648	0.8930	0.6989	0.9037
$Q_2$, $Q_1$, $P_2$, $P_1$	0.4495	0.5065	0.6446	0.9015	0.8734	0.9355
$Q_2$, $P_3$, $P_2$, $P_1$	0.4639	0.5040	0.6088	0.7818	0.8452	0.9045
$Q_1$, $P_3$, $P_2$, $P_1$	0.4415	0.5070	0.6326	0.8912	0.8458	0.9335
$Q_3$, $Q_2$, $Q_1$, $P_3$, $P_2$	0.4859	0.5044	0.5277	0.8919	0.6246	0.8911
$Q_3$, $Q_2$, $Q_1$, $P_3$, $P_1$	0.4646	0.4972	0.5655	0.8956	0.7031	0.9073
$Q_3$, $Q_2$, $Q_1$, $P_2$, $P_1$	0.5008	0.4988	0.6065	0.8996	0.8332	0.9351
$Q_3$, $Q_2$, $P_3$, $P_2$, $P_1$	0.4528	0.5127	0.5701	0.7641	0.8577	0.8996
$Q_3$, $Q_1$, $P_3$, $P_2$, $P_1$	0.5331	0.5060	0.6081	0.8892	0.8406	0.9334
$Q_2$, $Q_1$, $P_3$, $P_2$, $P_1$	0.4984	0.5101	0.5974	0.9002	0.8442	0.9370
$Q_3$, $Q_2$, $Q_1$, $P_3$, $P_2$, $P_1$	0.4155	0.4986	0.5800	0.8984	0.7703	0.9325

## SAS combination search results

The $F_1$ scores, sensitivities, and specificities achieved for SAS classification when using each of the six ML methods are shown in Tables 19, 20, and 21, respectively.

**Table 19:** The $F_1$ scores achieved across the combination search by each of the six classification methods

Input combination	Classification method					
	NB	LR	SVM	RF	MLP	GB
$Q_3$	0.5041	0.5288	0.4897	0.5723	0.5403	0.5592
$Q_2$	0.4681	0.5004	0.4839	0.7577	0.5691	0.7415
$Q_1$	0.3799	0.5028	0.4923	0.7779	0.6176	0.7529
$P_3$	0.4931	0.4972	0.5097	0.5530	0.5474	0.5331
$P_2$	0.4698	0.4990	0.5528	0.5627	0.4895	0.5453
$P_1$	0.5344	0.5023	0.5035	0.5171	0.5571	0.5060
$Q_3$, $Q_2$	0.4529	0.5136	0.5075	0.7623	0.4939	0.7608
$Q_3$, $Q_1$	0.4588	0.4893	0.5053	0.7814	0.5414	0.7758
$Q_3$, $P_3$	0.4992	0.4963	0.5207	0.5824	0.5463	0.5746
$Q_3$, $P_2$	0.5497	0.5068	0.5306	0.5869	0.5215	0.5850
$Q_3$, $P_1$	0.4195	0.5099	0.4992	0.5685	0.4776	0.5627
$Q_2$, $Q_1$	0.5064	0.5010	0.5025	0.8450	0.5853	0.8461
$Q_2$, $P_3$	0.4818	0.5020	0.5294	0.7555	0.6054	0.7694
$Q_2$, $P_2$	0.5116	0.5020	0.5405	0.7586	0.5454	0.7711
$Q_2$, $P_1$	0.5468	0.4913	0.5353	0.7568	0.5124	0.7609
$Q_1$, $P_3$	0.4564	0.4963	0.5252	0.7697	0.5067	0.7522
$Q_1$, $P_2$	0.5209	0.4986	0.5388	0.7708	0.5833	0.7606
$Q_1$, $P_1$	0.5186	0.5005	0.5327	0.7744	0.5426	0.7751
$P_3$, $P_2$	0.5450	0.5031	0.5256	0.5695	0.4960	0.5626
$P_3$, $P_1$	0.5464	0.4996	0.5282	0.5450	0.5510	0.5338
$P_2$, $P_1$	0.5399	0.5041	0.5447	0.5669	0.5133	0.5766
$Q_3$, $Q_2$, $Q_1$	0.4574	0.5081	0.5284	0.8447	0.5866	0.8552
$Q_3$, $Q_2$, $P_3$	0.5499	0.4925	0.5254	0.7624	0.5847	0.7830
$Q_3$, $Q_2$, $P_2$	0.4591	0.4936	0.5272	0.7629	0.5742	0.7829
$Q_3$, $Q_2$, $P_1$	0.4240	0.4980	0.5099	0.7627	0.4969	0.7800
$Q_3$, $Q_1$, $P_3$	0.4810	0.4994	0.5173	0.7808	0.5511	0.7691
$Q_3$, $Q_1$, $P_2$	0.4098	0.5069	0.5354	0.7749	0.5611	0.7750
$Q_3$, $Q_1$, $P_1$	0.5414	0.4999	0.5095	0.7761	0.5230	0.7880
$Q_3$, $P_3$, $P_2$	0.4492	0.5021	0.5330	0.5892	0.5636	0.5900
$Q_3$, $P_3$, $P_1$	0.4912	0.4971	0.5248	0.5767	0.5253	0.5759
$Q_3$, $P_2$, $P_1$	0.4476	0.4914	0.5259	0.5883	0.5758	0.5961
$Q_2$, $Q_1$, $P_3$	0.5243	0.5008	0.5154	0.8381	0.5874	0.8427
$Q_2$, $Q_1$, $P_2$	0.4994	0.5029	0.5349	0.8402	0.6139	0.8469
$Q_2$, $Q_1$, $P_1$	0.4988	0.5042	0.5279	0.8413	0.5861	0.8492
$Q_2$, $P_3$, $P_2$	0.5272	0.4992	0.5284	0.7549	0.5760	0.7802
$Q_2$, $P_3$, $P_1$	0.4351	0.5048	0.5351	0.7479	0.5724	0.7726
$Q_2$, $P_2$, $P_1$	0.5318	0.5081	0.5316	0.7563	0.5258	0.7752
$Q_1$, $P_3$, $P_2$	0.5152	0.5030	0.5454	0.7624	0.5782	0.7579
$Q_1$, $P_3$, $P_1$	0.4607	0.5022	0.5235	0.7690	0.5069	0.7680
$Q_1$, $P_2$, $P_1$	0.5437	0.5019	0.5319	0.7670	0.5930	0.7733
$P_3$, $P_2$, $P_1$	0.5314	0.4984	0.5352	0.5661	0.5518	0.5826
$Q_3$, $Q_2$, $Q_1$, $P_3$	0.4910	0.4925	0.5169	0.8407	0.5706	0.8541
$Q_3$, $Q_2$, $Q_1$, $P_2$	0.5113	0.5036	0.5301	0.8432	0.5952	0.8585
$Q_3$, $Q_2$, $Q_1$, $P_1$	0.5097	0.5078	0.5191	0.8404	0.5828	0.8558
$Q_3$, $Q_2$, $P_3$, $P_2$	0.4738	0.4968	0.5206	0.7549	0.5628	0.7879
$Q_3$, $Q_2$, $P_3$, $P_1$	0.4721	0.4944	0.5224	0.7545	0.5605	0.7857
$Q_3$, $Q_2$, $P_2$, $P_1$	0.5592	0.5081	0.5331	0.7616	0.5854	0.7911
$Q_3$, $Q_1$, $P_3$, $P_2$	0.4762	0.4987	0.5259	0.7738	0.5791	0.7711
$Q_3$, $Q_1$, $P_3$, $P_1$	0.4558	0.5108	0.5339	0.7749	0.5766	0.7850
$Q_3$, $Q_1$, $P_2$, $P_1$	0.4066	0.4957	0.5279	0.7719	0.5785	0.7813
$Q_3$, $P_3$, $P_2$, $P_1$	0.5257	0.4878	0.5395	0.5866	0.5695	0.5988
$Q_2$, $Q_1$, $P_3$, $P_2$	0.5318	0.4975	0.5487	0.8357	0.6064	0.8488
$Q_2$, $Q_1$, $P_3$, $P_1$	0.5348	0.4987	0.5326	0.8350	0.5879	0.8516
$Q_2$, $Q_1$, $P_2$, $P_1$	0.5537	0.5113	0.5337	0.8362	0.6258	0.8545
$Q_2$, $P_3$, $P_2$, $P_1$	0.4863	0.4966	0.5394	0.7458	0.6102	0.7797
$Q_1$, $P_3$, $P_2$, $P_1$	0.4711	0.5010	0.5358	0.7635	0.6088	0.7738
$Q_3$, $Q_2$, $Q_1$, $P_3$, $P_2$	0.4763	0.5038	0.5312	0.8330	0.5966	0.8534
$Q_3$, $Q_2$, $Q_1$, $P_3$, $P_1$	0.4953	0.4998	0.5212	0.8399	0.5809	0.8571
$Q_3$, $Q_2$, $Q_1$, $P_2$, $P_1$	0.4917	0.5099	0.5304	0.8390	0.6070	0.8600
$Q_3$, $Q_2$, $P_3$, $P_2$, $P_1$	0.5344	0.5069	0.5292	0.7540	0.5963	0.7913
$Q_3$, $Q_1$, $P_3$, $P_2$, $P_1$	0.5205	0.4991	0.5309	0.7734	0.5740	0.7828
$Q_2$, $Q_1$, $P_3$, $P_2$, $P_1$	0.4912	0.5012	0.5353	0.8325	0.6302	0.8502
$Q_3$, $Q_2$, $Q_1$, $P_3$, $P_2$, $P_1$	0.4642	0.5016	0.5301	0.8292	0.6040	0.8574

**Table 20:** The sensitivities achieved across the combination search by each of the six classification methods

Input combination	Classification method					
	NB	LR	SVM	RF	MLP	GB
$Q_3$	0.2997	0.4576	0.5129	0.5678	0.4059	0.5585
$Q_2$	0.5460	0.5348	0.6918	0.7517	0.3839	0.7366
$Q_1$	0.7074	0.4613	0.6338	0.7582	0.1873	0.7224
$P_3$	0.4402	0.5127	0.5616	0.5453	0.3978	0.5431
$P_2$	0.5140	0.4981	0.4783	0.5629	0.5704	0.5717
$P_1$	0.4446	0.4836	0.4741	0.5177	0.3803	0.5244
$Q_3$, $Q_2$	0.5683	0.4928	0.5411	0.7612	0.5901	0.7585
$Q_3$, $Q_1$	0.4887	0.4947	0.5036	0.7630	0.4709	0.7504
$Q_3$, $P_3$	0.6479	0.5019	0.5147	0.5720	0.4578	0.5808
$Q_3$, $P_2$	0.5719	0.4985	0.5163	0.5849	0.5223	0.5999
$Q_3$, $P_1$	0.6081	0.4947	0.4958	0.5633	0.5570	0.5788
$Q_2$, $Q_1$	0.6572	0.5008	0.6082	0.8374	0.4909	0.8293
$Q_2$, $P_3$	0.5785	0.4860	0.5626	0,.7505	0.4320	0.7710
$Q_2$, $P_2$	0.4241	0.4801	0.5294	0.7560	0.6660	0.7763
$Q_2$, $P_1$	0.2405	0.5006	0.5127	0.7500	0.5838	0.7601
$Q_1$, $P_3$	0.5330	0.4970	0.5596	0.7534	0.5809	0.7305
$Q_1$, $P_2$	0.4943	0.5180	0.5282	0.7545	0.4884	0.7434
$Q_1$, $P_1$	0.5761	0.4991	0.5430	0.7516	0.6004	0.7549
$P_3$, $P_2$	0.4714	0.4939	0.5388	0.5668	0.6677	0.5744
$P_3$, $P_1$	0.5408	0.4954	0.5252	0.5406	0.4456	0.5421
$P_2$, $P_1$	0.4115	0.4958	0.4761	0.5761	0.6175	0.6056
$Q_3$, $Q_2$, $Q_1$	0.5695	0.5019	0.5106	0.8271	0.5303	0.8453
$Q_3$, $Q_2$, $P_3$	0.5651	0.5115	0.5075	0.7621	0.5044	0.7826
$Q_3$, $Q_2$, $P_2$	0.5768	0.5219	0.5101	0.7590	0.5941	0.7882
$Q_3$, $Q_2$, $P_1$	0.6416	0.5013	0.5350	0.7494	0.5963	0.7766
$Q_3$, $Q_1$, $P_3$	0.4649	0.5074	0.5237	0.7550	0.5783	0.7491
$Q_3$, $Q_1$, $P_2$	0.6031	0.50	0.5056	0.7584	0.5796	0.7514
$Q_3$, $Q_1$, $P_1$	0.3262	0.4942	0.5535	0.7527	0.6028	0.7677
$Q_3$, $P_3$, $P_2$	0.5316	0.4904	0.5184	0.5924	0.4985	0.6109
$Q_3$, $P_3$, $P_1$	0.3543	0.4949	0.5116	0.5765	0.5444	0.5855
$Q_3$, $P_2$, $P_1$	0.5225	0.5038	0.5041	0.5864	0.5018	0.6186
$Q_2$, $Q_1$, $P_3$	0.4531	0.4826	0.5427	0.8186	0.6309	0.8303
$Q_2$, $Q_1$, $P_2$	0.4642	0.5029	0.5481	0.8277	0.6178	0.8312
$Q_2$, $Q_1$, $P_1$	0.5179	0.5049	0.5544	0.8268	0.5788	0.8388
$Q_2$, $P_3$, $P_2$	0.5155	0.4806	0.5642	0.7500	0.6050	0.7757
$Q_2$, $P_3$, $P_1$	0.6119	0.4972	0.5365	0.7486	0.5358	0.7752
$Q_2$, $P_2$, $P_1$	0.5590	0.5214	0.5403	0.7578	0.7119	0.7791
$Q_1$, $P_3$, $P_2$	0.4890	0.5159	0.5345	0.7414	0.5886	0.7437
$Q_1$, $P_3$, $P_1$	0.5256	0.5041	0.5548	0.7498	0.6421	0.7479
$Q_1$, $P_2$, $P_1$	0.4038	0.5014	0.5175	0.7490	0.5995	0.7621
$P_3$, $P_2$, $P_1$	0.4461	0.4995	0.5216	0.5697	0.6360	0.6026
$Q_3$, $Q_2$, $Q_1$, $P_3$	0.6262	0.5155	0.5310	0.8274	0.6144	0.8411
$Q_3$, $Q_2$, $Q_1$, $P_2$	0.4646	0.5158	0.5531	0.8303	0.6113	0.8487
$Q_3$, $Q_2$, $Q_1$, $P_1$	0.4913	0.5011	0.5522	0.8242	0.5723	0.8466
$Q_3$, $Q_2$, $P_3$, $P_2$	0.5435	0.4831	0.54	0.7566	0.64	0.7924
$Q_3$, $Q_2$, $P_3$, $P_1$	0.5466	0.4884	0.5173	0.7534	0.5521	0.7874
$Q_3$, $Q_2$, $P_2$, $P_1$	0.4776	0.5022	0.5413	0.7555	0.5892	0.7900
$Q_3$, $Q_1$, $P_3$, $P_2$	0.5274	0.5010	0.5377	0.7587	0.5758	0.7545
$Q_3$, $Q_1$, $P_3$, $P_1$	0.4177	0.4823	0.5051	0.7560	0.5163	0.7675
$Q_3$, $Q_1$, $P_2$, $P_1$	0.5806	0.5103	0.5087	0.7550	0.5940	0.7735
$Q_3$, $P_3$, $P_2$, $P_1$	0.46	0.5052	0.5204	0.5857	0.6047	0.6121
$Q_2$, $Q_1$, $P_3$, $P_2$	0.4529	0.5117	0.5461	0.8241	0.6431	0.8413
$Q_2$, $Q_1$, $P_3$, $P_1$	0.2714	0.4964	0.5150	0.8186	0.6153	0.8437
$Q_2$, $Q_1$, $P_2$, $P_1$	0.5132	0.5057	0.5357	0.8214	0.6157	0.8386
$Q_2$, $P_3$, $P_2$, $P_1$	0.4464	0.5042	0.5606	0.7407	0.6294	0.7833
$Q_1$, $P_3$, $P_2$, $P_1$	0.4715	0.5032	0.5476	0.7439	0.6014	0.7599
$Q_3$, $Q_2$, $Q_1$, $P_3$, $P_2$	0.44	0.4889	0.5266	0.8175	0.5881	0.8510
$Q_3$, $Q_2$, $Q_1$, $P_3$, $P_1$	0.3896	0.4988	0.5447	0.8256	0.6080	0.8443
$Q_3$, $Q_2$, $Q_1$, $P_2$, $P_1$	0.5676	0.50	0.5270	0.8274	0.6084	0.8525
$Q_3$, $Q_2$, $P_3$, $P_2$, $P_1$	0.4376	0.5137	0.5454	0.7499	0.6264	0.7859
$Q_3$, $Q_1$, $P_3$, $P_2$, $P_1$	0.4463	0.4941	0.5332	0.7509	0.6137	0.7634
$Q_2$, $Q_1$, $P_3$, $P_2$, $P_1$	0.6175	0.4940	0.5561	0.8159	0.5996	0.8451
$Q_3$, $Q_2$, $Q_1$, $P_3$, $P_2$, $P_1$	0.5047	0.4996	0.5342	0.8102	0.6133	0.8504

**Table 21:** The specificities achieved across the combination search by each of the six classification methods

Input combination	Classification method					
	NB	LR	SVM	RF	MLP	GB
$Q_3$	0.5731	0.5544	0.4823	0.5742	0.5901	0.5596
$Q_2$	0.4444	0.4890	0.4176	0.7615	0.6429	0.7445
$Q_1$	0.3032	0.5168	0.4462	0.7905	0.8099	0.7714
$P_3$	0.5105	0.4921	0.4920	0.5560	0.6038	0.5295
$P_2$	0.4563	0.4993	0.5814	0.5627	0.4633	0.5355
$P_1$	0.5672	0.5087	0.5135	0.5170	0.6254	0.4999
$Q_3$, $Q_2$	0.4192	0.5208	0.4962	0.7631	0.4624	0.7623
$Q_3$, $Q_1$	0.4499	0.4876	0.5059	0.7932	0.5676	0.7920
$Q_3$, $P_3$	0.4498	0.4945	0.5229	0.5867	0.5796	0.5722
$Q_3$, $P_2$	0.5414	0.5097	0.5359	0.5878	0.5213	0.5789
$Q_3$, $P_1$	0.3695	0.5152	0.5004	0.5706	0.4528	0.5565
$Q_2$, $Q_1$	0.4553	0.5012	0.4671	0.8507	0.6244	0.8585
$Q_2$, $P_3$	0.4512	0.5074	0.5175	0.7586	0.6807	0.7685
$Q_2$, $P_2$	0.5418	0.5094	0.5447	0.7603	0.5002	0.7679
$Q_2$, $P_1$	0.6622	0.4884	0.5436	0.7610	0.4879	0.7614
$Q_1$, $P_3$	0.4338	0.4961	0.5130	0.7800	0.4816	0.7653
$Q_1$, $P_2$	0.5303	0.4922	0.5428	0.7811	0.6224	0.7712
$Q_1$, $P_1$	0.4985	0.5010	0.5290	0.7889	0.5211	0.7880
$P_3$, $P_2$	0.5726	0.5062	0.5209	0.5707	0.4394	0.5581
$P_3$, $P_1$	0.5486	0.5011	0.5293	0.5467	0.5912	0.5309
$P_2$, $P_1$	0.5874	0.5069	0.5704	0.5633	0.4774	0.5649
$Q_3$, $Q_2$, $Q_1$	0.4242	0.5103	0.5348	0.8576	0.6101	0.8626
$Q_3$, $Q_2$, $P_3$	0.5442	0.4864	0.5319	0.7626	0.6180	0.7834
$Q_3$, $Q_2$, $P_2$	0.4241	0.4844	0.5334	0.7654	0.5662	0.7795
$Q_3$, $Q_2$, $P_1$	0.3655	0.4970	0.5014	0.7710	0.4641	0.7822
$Q_3$, $Q_1$, $P_3$	0.4862	0.4968	0.5152	0.7974	0.5408	0.7816
$Q_3$, $Q_1$, $P_2$	0.3600	0.5093	0.5464	0.7854	0.5539	0.7900
$Q_3$, $Q_1$, $P_1$	0.6213	0.5018	0.4945	0.7911	0.4948	0.8013
$Q_3$, $P_3$, $P_2$	0.4254	0.5061	0.5384	0.5879	0.5892	0.5813
$Q_3$, $P_3$, $P_1$	0.5358	0.4979	0.5295	0.5769	0.5186	0.5721
$Q_3$, $P_2$, $P_1$	0.4261	0.4874	0.5338	0.5892	0.6058	0.5866
$Q_2$, $Q_1$, $P_3$	0.5497	0.5069	0.5060	0.8522	0.5694	0.8519
$Q_2$, $Q_1$, $P_2$	0.5112	0.5029	0.5301	0.8494	0.6123	0.8585
$Q_2$, $Q_1$, $P_1$	0.4925	0.5040	0.5184	0.8519	0.5892	0.8569
$Q_2$, $P_3$, $P_2$	0.5315	0.5055	0.5156	0.7579	0.5643	0.7831
$Q_2$, $P_3$, $P_1$	0.3860	0.5075	0.5347	0.7476	0.5871	0.7710
$Q_2$, $P_2$, $P_1$	0.5220	0.5036	0.5285	0.7555	0.4595	0.7728
$Q_1$, $P_3$, $P_2$	0.5244	0.4987	0.5495	0.7755	0.5740	0.7667
$Q_1$, $P_3$, $P_1$	0.4414	0.5016	0.5125	0.7810	0.4611	0.7806
$Q_1$, $P_2$, $P_1$	0.5960	0.5021	0.5372	0.7782	0.5904	0.7804
$P_3$, $P_2$, $P_1$	0.5624	0.4981	0.5403	0.5647	0.5198	0.5745
$Q_3$, $Q_2$, $Q_1$, $P_3$	0.4471	0.4850	0.5120	0.8504	0.5532	0.8638
$Q_3$, $Q_2$, $Q_1$, $P_2$	0.5274	0.4996	0.5219	0.8527	0.5884	0.8660
$Q_3$, $Q_2$, $Q_1$, $P_1$	0.5160	0.5102	0.5076	0.8522	0.5872	0.8627
$Q_3$, $Q_2$, $P_3$, $P_2$	0.4522	0.5014	0.5138	0.7540	0.5326	0.7851
$Q_3$, $Q_2$, $P_3$, $P_1$	0.4492	0.4964	0.5243	0.7553	0.5639	0.7847
$Q_3$, $Q_2$, $P_2$, $P_1$	0.5909	0.5102	0.5302	0.7654	0.5839	0.7919
$Q_3$, $Q_1$, $P_3$, $P_2$	0.4603	0.4980	0.5218	0.7834	0.5805	0.7816
$Q_3$, $Q_1$, $P_3$, $P_1$	0.4671	0.5206	0.5444	0.7869	0.6011	0.7964
$Q_3$, $Q_1$, $P_2$, $P_1$	0.3623	0.4910	0.5348	0.7826	0.5723	0.7864
$Q_3$, $P_3$, $P_2$, $P_1$	0.5492	0.4823	0.5466	0.5870	0.5555	0.5932
$Q_2$, $Q_1$, $P_3$, $P_2$	0.5604	0.4929	0.5497	0.8441	0.5905	0.8545
$Q_2$, $Q_1$, $P_3$, $P_1$	0.6311	0.4995	0.5390	0.8468	0.5765	0.8575
$Q_2$, $Q_1$, $P_2$, $P_1$	0.5693	0.5133	0.5330	0.8469	0.6304	0.8664
$Q_2$, $P_3$, $P_2$, $P_1$	0.4992	0.4941	0.5316	0.7489	0.6018	0.7774
$Q_1$, $P_3$, $P_2$, $P_1$	0.4710	0.5003	0.5315	0.7757	0.6121	0.7826
$Q_3$, $Q_2$, $Q_1$, $P_3$, $P_2$	0.4877	0.5089	0.5329	0.8442	0.6003	0.8552
$Q_3$, $Q_2$, $Q_1$, $P_3$, $P_1$	0.5301	0.5002	0.5130	0.8504	0.5699	0.8668
$Q_3$, $Q_2$, $Q_1$, $P_2$, $P_1$	0.4670	0.5133	0.5317	0.8475	0.6064	0.8657
$Q_3$, $Q_2$, $P_3$, $P_2$, $P_1$	0.5697	0.5046	0.5234	0.7566	0.5836	0.7950
$Q_3$, $Q_1$, $P_3$, $P_2$, $P_1$	0.5467	0.5008	0.5302	0.7876	0.5581	0.7954
$Q_2$, $Q_1$, $P_3$, $P_2$, $P_1$	0.4502	0.5037	0.5278	0.8444	0.6443	0.8540
$Q_3$, $Q_2$, $Q_1$, $P_3$, $P_2$, $P_1$	0.4520	0.5023	0.5287	0.8427	0.60	0.8627

## PAD combination search results

The $F_1$ scores, sensitivities, and specificities achieved for PAD classification when using each of the six ML methods are shown in Tables 22, 23, and 24 respectively.

**Table 22:** The $F_1$ scores achieved across the combination search by each of the six classification methods

Input combination	Classification method					
	NB	LR	SVM	RF	MLP	GB
$Q_3$	0.5017	0.5115	0.6645	0.8224	0.6897	0.8169
$Q_2$	0.5621	0.5222	0.5266	0.7127	0.4734	0.7076
$Q_1$	0.3927	0.4822	0.5310	0.8240	0.4713	0.8183
$P_3$	0.5162	0.5053	0.5182	0.5613	0.4131	0.5406
$P_2$	0.5030	0.4954	0.5242	0.5753	0.4741	0.5529
$P_1$	0.4290	0.5031	0.5038	0.5517	0.5487	0.5335
$Q_3$, $Q_2$	0.4740	0.5099	0.5926	0.8480	0.7040	0.8557
$Q_3$, $Q_1$	0.5355	0.4965	0.5786	0.8959	0.7254	0.9041
$Q_3$, $P_3$	0.4800	0.4932	0.5808	0.8050	0.6676	0.8151
$Q_3$, $P_2$	0.5118	0.4998	0.5824	0.8152	0.7057	0.8201
$Q_3$, $P_1$	0.5672	0.4979	0.5768	0.8103	0.7206	0.8221
$Q_2$, $Q_1$	0.5236	0.4962	0.5239	0.8556	0.5610	0.8637
$Q_2$, $P_3$	0.4929	0.4980	0.5069	0.7134	0.6117	0.7200
$Q_2$, $P_2$	0.5323	0.4956	0.5133	0.7126	0.5233	0.7255
$Q_2$, $P_1$	0.4602	0.5075	0.5222	0.7117	0.5585	0.7221
$Q_1$, $P_3$	0.5293	0.5116	0.5420	0.8136	0.5602	0.8204
$Q_1$, $P_2$	0.5335	0.4926	0.5406	0.8187	0.5818	0.8314
$Q_1$, $P_1$	0.5549	0.5011	0.5417	0.8181	0.6514	0.8307
$P_3$, $P_2$	0.4829	0.4996	0.5319	0.5810	0.5386	0.5733
$P_3$, $P_1$	0.4823	0.4976	0.5142	0.5624	0.5141	0.5559
$P_2$, $P_1$	0.5434	0.5035	0.5145	0.5904	0.4662	0.6002
$Q_3$, $Q_2$, $Q_1$	0.5209	0.4891	0.5619	0.9061	0.7004	0.9168
$Q_3$, $Q_2$, $P_3$	0.4717	0.5146	0.5605	0.8370	0.6864	0.8556
$Q_3$, $Q_2$, $P_2$	0.4651	0.5049	0.5640	0.8424	0.7074	0.8606
$Q_3$, $Q_2$, $P_1$	0.4643	0.5064	0.5610	0.8408	0.7040	0.8592
$Q_3$, $Q_1$, $P_3$	0.4947	0.4976	0.5679	0.8833	0.7148	0.9009
$Q_3$, $Q_1$, $P_2$	0.5615	0.4984	0.5741	0.8858	0.7100	0.9022
$Q_3$, $Q_1$, $P_1$	0.4149	0.4941	0.5760	0.8850	0.7361	0.9046
$Q_3$, $P_3$, $P_2$	0.4800	0.5065	0.5598	0.8005	0.6804	0.8215
$Q_3$, $P_3$, $P_1$	0.5214	0.5050	0.5642	0.8005	0.6886	0.8179
$Q_3$, $P_2$, $P_1$	0.4792	0.5065	0.5630	0.8004	0.7104	0.8178
$Q_2$, $Q_1$, $P_3$	0.5208	0.5006	0.5334	0.8469	0.6300	0.8617
$Q_2$, $Q_1$, $P_2$	0.4874	0.4974	0.5318	0.8472	0.5992	0.8703
$Q_2$, $Q_1$, $P_1$	0.5340	0.4938	0.5311	0.8472	0.6472	0.8682
$Q_2$, $P_3$, $P_2$	0.5306	0.4996	0.5162	0.7147	0.5581	0.7379
$Q_2$, $P_3$, $P_1$	0.5012	0.4989	0.5152	0.7062	0.5165	0.7311
$Q_2$, $P_2$, $P_1$	0.5165	0.4983	0.5232	0.7118	0.5659	0.7322
$Q_1$, $P_3$, $P_2$	0.5324	0.4941	0.5382	0.8086	0.6117	0.8302
$Q_1$, $P_3$, $P_1$	0.4632	0.5047	0.5322	0.8116	0.6127	0.8324
$Q_1$, $P_2$, $P_1$	0.4524	0.4930	0.5429	0.8146	0.6441	0.8380
$P_3$, $P_2$, $P_1$	0.5016	0.5023	0.5262	0.5838	0.5654	0.6078
$Q_3$, $Q_2$, $Q_1$, $P_3$	0.5480	0.5086	0.5600	0.8992	0.6988	0.9138
$Q_3$, $Q_2$, $Q_1$, $P_2$	0.4505	0.4997	0.5564	0.8997	0.7017	0.9164
$Q_3$, $Q_2$, $Q_1$, $P_1$	0.4973	0.5053	0.5601	0.8990	0.7030	0.9196
$Q_3$, $Q_2$, $P_3$, $P_2$	0.3998	0.4993	0.5601	0.8376	0.6688	0.8612
$Q_3$, $Q_2$, $P_3$, $P_1$	0.5253	0.4973	0.5558	0.8330	0.6738	0.8556
$Q_3$, $Q_2$, $P_2$, $P_1$	0.4726	0.4972	0.5650	0.8385	0.6811	0.8597
$Q_3$, $Q_1$, $P_3$, $P_2$	0.5030	0.4976	0.5684	0.8803	0.6845	0.8999
$Q_3$, $Q_1$, $P_3$, $P_1$	0.5189	0.5019	0.5595	0.8839	0.6849	0.9013
$Q_3$, $Q_1$, $P_2$, $P_1$	0.5692	0.4994	0.5715	0.8805	0.6962	0.9025
$Q_3$, $P_3$, $P_2$, $P_1$	0.4801	0.4991	0.5576	0.7940	0.6746	0.8170
$Q_2$, $Q_1$, $P_3$, $P_2$	0.4681	0.4966	0.5404	0.8417	0.6239	0.8624
$Q_2$, $Q_1$, $P_3$, $P_1$	0.5009	0.5015	0.5278	0.8378	0.6146	0.8677
$Q_2$, $Q_1$, $P_2$, $P_1$	0.5278	0.4979	0.5304	0.8433	0.6327	0.8690
$Q_2$, $P_3$, $P_2$, $P_1$	0.5242	0.5024	0.5180	0.7022	0.5806	0.7376
$Q_1$, $P_3$, $P_2$, $P_1$	0.4996	0.5033	0.5355	0.8087	0.6158	0.8328
$Q_3$, $Q_2$, $Q_1$, $P_3$, $P_2$	0.5012	0.5006	0.5495	0.8971	0.6889	0.9169
$Q_3$, $Q_2$, $Q_1$, $P_3$, $P_1$	0.5025	0.4969	0.5562	0.8952	0.6887	0.9151
$Q_3$, $Q_2$, $Q_1$, $P_2$, $P_1$	0.5023	0.5019	0.5502	0.8969	0.6895	0.9170
$Q_3$, $Q_2$, $P_3$, $P_2$, $P_1$	0.4946	0.4923	0.5488	0.8279	0.6545	0.8597
$Q_3$, $Q_1$, $P_3$, $P_2$, $P_1$	0.4489	0.4972	0.5666	0.8758	0.6688	0.9042
$Q_2$, $Q_1$, $P_3$, $P_2$, $P_1$	0.5377	0.4995	0.5391	0.8389	0.6154	0.8655
$Q_3$, $Q_2$, $Q_1$, $P_3$, $P_2$, $P_1$	0.4479	0.4974	0.5573	0.8935	0.6681	0.9187

**Table 23:** The sensitivities achieved across the combination search by each of the six classification methods

Input combination	Classification method					
	NB	LR	SVM	RF	MLP	GB
$Q_3$	0.3598	0.5048	0.6806	0.8219	0.5998	0.8188
$Q_2$	0.5441	0.4878	0.5879	0.6858	0.5536	0.6922
$Q_1$	0.5735	0.5026	0.6065	0.8126	0.5959	0.8140
$P_3$	0.4246	0.4935	0.5472	0.5358	0.6388	0.5425
$P_2$	0.4565	0.4985	0.5368	0.5532	0.5572	0.5576
$P_1$	0.6253	0.5001	0.5571	0.5245	0.3899	0.5261
$Q_3$, $Q_2$	0.5595	0.4912	0.6297	0.8414	0.7176	0.8532
$Q_3$, $Q_1$	0.4753	0.5087	0.6324	0.8825	0.7460	0.8950
$Q_3$, $P_3$	0.6086	0.5025	0.5980	0.8021	0.6523	0.8173
$Q_3$, $P_2$	0.3310	0.4895	0.5919	0.8089	0.7679	0.8269
$Q_3$, $P_1$	0.3079	0.5280	0.6021	0.8051	0.7461	0.8266
$Q_2$, $Q_1$	0.4323	0.4902	0.5878	0.8346	0.6016	0.8521
$Q_2$, $P_3$	0.5419	0.4877	0.5744	0.6826	0.2813	0.7126
$Q_2$, $P_2$	0.5505	0.5051	0.5776	0.6862	0.5169	0.7275
$Q_2$, $P_1$	0.6100	0.4976	0.5697	0.6875	0.4716	0.7127
$Q_1$, $P_3$	0.3309	0.4971	0.5476	0.8001	0.5911	0.8168
$Q_1$, $P_2$	0.5495	0.5063	0.5827	0.8019	0.5508	0.8288
$Q_1$, $P_1$	0.3834	0.4930	0.5778	0.8059	0.6787	0.8272
$P_3$, $P_2$	0.4789	0.4946	0.5458	0.5569	0.5443	0.5709
$P_3$, $P_1$	0.5309	0.5066	0.5642	0.5425	0.5406	0.5484
$P_2$, $P_1$	0.5325	0.4961	0.5863	0.5651	0.6096	0.5998
$Q_3$, $Q_2$, $Q_1$	0.4948	0.5163	0.5976	0.8885	0.7801	0.9055
$Q_3$, $Q_2$, $P_3$	0.3895	0.4985	0.5568	0.8323	0.7286	0.8572
$Q_3$, $Q_2$, $P_2$	0.5612	0.5051	0.5851	0.8388	0.6953	0.8545
$Q_3$, $Q_2$, $P_1$	0.4521	0.4890	0.5787	0.8278	0.7259	0.8559
$Q_3$, $Q_1$, $P_3$	0.5637	0.5045	0.5826	0.8707	0.7050	0.8913
$Q_3$, $Q_1$, $P_2$	0.4240	0.5030	0.5974	0.8710	0.7409	0.8923
$Q_3$, $Q_1$, $P_1$	0.6578	0.5094	0.6104	0.8663	0.6902	0.8928
$Q_3$, $P_3$, $P_2$	0.3869	0.4995	0.5834	0.7984	0.6967	0.8211
$Q_3$, $P_3$, $P_1$	0.2820	0.5009	0.5706	0.7914	0.6994	0.8208
$Q_3$, $P_2$, $P_1$	0.5814	0.4880	0.5824	0.7970	0.6789	0.8163
$Q_2$, $Q_1$, $P_3$	0.3260	0.4775	0.5663	0.8303	0.5969	0.8540
$Q_2$, $Q_1$, $P_2$	0.4239	0.4959	0.5625	0.8309	0.6028	0.8636
$Q_2$, $Q_1$, $P_1$	0.3205	0.5176	0.5610	0.8289	0.6418	0.8595
$Q_2$, $P_3$, $P_2$	0.4276	0.4900	0.5714	0.6920	0.5968	0.7328
$Q_2$, $P_3$, $P_1$	0.5554	0.4896	0.5560	0.6859	0.6252	0.7136
$Q_2$, $P_2$, $P_1$	0.4250	0.5134	0.5664	0.6845	0.5546	0.7245
$Q_1$, $P_3$, $P_2$	0.5668	0.4987	0.5330	0.7935	0.5752	0.8208
$Q_1$, $P_3$, $P_1$	0.4876	0.5104	0.5537	0.7998	0.6082	0.8287
$Q_1$, $P_2$, $P_1$	0.6109	0.4885	0.5572	0.8022	0.5978	0.8313
$P_3$, $P_2$, $P_1$	0.3959	0.4901	0.5652	0.5688	0.5532	0.6022
$Q_3$, $Q_2$, $Q_1$, $P_3$	0.3678	0.4879	0.5510	0.8819	0.7136	0.9035
$Q_3$, $Q_2$, $Q_1$, $P_2$	0.4522	0.5111	0.5909	0.8868	0.7224	0.9085
$Q_3$, $Q_2$, $Q_1$, $P_1$	0.5593	0.4867	0.5680	0.8846	0.7250	0.9068
$Q_3$, $Q_2$, $P_3$, $P_2$	0.5688	0.4972	0.5879	0.8231	0.7166	0.8574
$Q_3$, $Q_2$, $P_3$, $P_1$	0.4517	0.5112	0.5707	0.8201	0.7036	0.8504
$Q_3$, $Q_2$, $P_2$, $P_1$	0.5414	0.4904	0.5642	0.8247	0.7091	0.8526
$Q_3$, $Q_1$, $P_3$, $P_2$	0.6603	0.4851	0.5512	0.8655	0.7055	0.8936
$Q_3$, $Q_1$, $P_3$, $P_1$	0.3708	0.4993	0.5781	0.8655	0.7178	0.8951
$Q_3$, $Q_1$, $P_2$, $P_1$	0.4094	0.4967	0.5752	0.8612	0.7042	0.8926
$Q_3$, $P_3$, $P_2$, $P_1$	0.5180	0.5097	0.5724	0.7834	0.6593	0.8182
$Q_2$, $Q_1$, $P_3$, $P_2$	0.3984	0.4901	0.5564	0.8199	0.6451	0.8568
$Q_2$, $Q_1$, $P_3$, $P_1$	0.3787	0.5159	0.5556	0.8243	0.6639	0.8587
$Q_2$, $Q_1$, $P_2$, $P_1$	0.4432	0.5153	0.5587	0.8324	0.6442	0.8633
$Q_2$, $P_3$, $P_2$, $P_1$	0.4612	0.4878	0.5385	0.6811	0.5837	0.7262
$Q_1$, $P_3$, $P_2$, $P_1$	0.4762	0.4917	0.5679	0.7953	0.6449	0.8315
$Q_3$, $Q_2$, $Q_1$, $P_3$, $P_2$	0.3675	0.5049	0.5659	0.8802	0.6844	0.9133
$Q_3$, $Q_2$, $Q_1$, $P_3$, $P_1$	0.3552	0.4925	0.5784	0.8766	0.6848	0.9073
$Q_3$, $Q_2$, $Q_1$, $P_2$, $P_1$	0.4635	0.4996	0.5754	0.8829	0.6910	0.9041
$Q_3$, $Q_2$, $P_3$, $P_2$, $P_1$	0.4797	0.5169	0.5518	0.8142	0.6891	0.8544
$Q_3$, $Q_1$, $P_3$, $P_2$, $P_1$	0.5274	0.5069	0.5507	0.8625	0.6738	0.8986
$Q_2$, $Q_1$, $P_3$, $P_2$, $P_1$	0.3947	0.4911	0.5493	0.8258	0.6190	0.8556
$Q_3$, $Q_2$, $Q_1$, $P_3$, $P_2$, $P_1$	0.6385	0.4859	0.5511	0.8813	0.6588	0.9102

**Table 24:** The specificities achieved across the combination search by each of the six classification methods

Input combination	Classification method					
	NB	LR	SVM	RF	MLP	GB
$Q_3$	0.5493	0.5139	0.6566	0.8228	0.7371	0.8157
$Q_2$	0.5692	0.5344	0.5047	0.7276	0.4486	0.7161
$Q_1$	0.3486	0.4758	0.5038	0.8320	0.4329	0.8214
$P_3$	0.5481	0.5093	0.5081	0.5713	0.3544	0.5399
$P_2$	0.5187	0.4945	0.5198	0.5843	0.4484	0.5512
$P_1$	0.3754	0.5042	0.4859	0.5622	0.6088	0.5362
$Q_3$, $Q_2$	0.4475	0.5164	0.5770	0.8529	0.6967	0.8576
$Q_3$, $Q_1$	0.5576	0.4926	0.5568	0.9068	0.7137	0.9117
$Q_3$, $P_3$	0.4395	0.4902	0.5738	0.8070	0.6754	0.8137
$Q_3$, $P_2$	0.5740	0.5033	0.5785	0.8196	0.6718	0.8155
$Q_3$, $P_1$	0.6699	0.4880	0.5666	0.8140	0.7063	0.8190
$Q_2$, $Q_1$	0.5561	0.4983	0.5013	0.8714	0.5452	0.8726
$Q_2$, $P_3$	0.4769	0.5015	0.4840	0.7305	0.7573	0.7243
$Q_2$, $P_2$	0.5257	0.4926	0.4912	0.7273	0.5257	0.7245
$Q_2$, $P_1$	0.4155	0.5109	0.5055	0.7252	0.5922	0.7275
$Q_1$, $P_3$	0.6008	0.5166	0.5400	0.8229	0.5482	0.8230
$Q_1$, $P_2$	0.5277	0.4882	0.5251	0.8305	0.5946	0.8334
$Q_1$, $P_1$	0.6209	0.5039	0.5284	0.8266	0.6383	0.8333
$P_3$, $P_2$	0.4842	0.5013	0.5269	0.5910	0.5365	0.5743
$P_3$, $P_1$	0.4669	0.4947	0.4970	0.5703	0.5050	0.5589
$P_2$, $P_1$	0.5476	0.5061	0.4897	0.6010	0.4227	0.6004
$Q_3$, $Q_2$, $Q_1$	0.5302	0.4803	0.5480	0.9208	0.6575	0.9265
$Q_3$, $Q_2$, $P_3$	0.4972	0.5203	0.5620	0.8405	0.6644	0.8545
$Q_3$, $Q_2$, $P_2$	0.4360	0.5049	0.5558	0.8451	0.7141	0.8653
$Q_3$, $Q_2$, $P_1$	0.4681	0.5123	0.5541	0.8504	0.6922	0.8618
$Q_3$, $Q_1$, $P_3$	0.4721	0.4954	0.5622	0.8933	0.7204	0.9088
$Q_3$, $Q_1$, $P_2$	0.6153	0.4970	0.5648	0.8976	0.6931	0.9104
$Q_3$, $Q_1$, $P_1$	0.3514	0.4892	0.5621	0.9000	0.7629	0.9144
$Q_3$, $P_3$, $P_2$	0.5095	0.5090	0.5507	0.8020	0.6720	0.8218
$Q_3$, $P_3$, $P_1$	0.6059	0.5064	0.5617	0.8066	0.6830	0.8159
$Q_3$, $P_2$, $P_1$	0.4470	0.5129	0.5555	0.8028	0.7279	0.8189
$Q_2$, $Q_1$, $P_3$	0.5894	0.5084	0.5215	0.8592	0.6453	0.8677
$Q_2$, $Q_1$, $P_2$	0.5079	0.4979	0.5207	0.8593	0.5977	0.8755
$Q_2$, $Q_1$, $P_1$	0.6118	0.4860	0.5203	0.8607	0.6498	0.8749
$Q_2$, $P_3$, $P_2$	0.5679	0.5029	0.4971	0.7274	0.5432	0.7410
$Q_2$, $P_3$, $P_1$	0.4831	0.5021	0.5011	0.7173	0.4787	0.7413
$Q_2$, $P_2$, $P_1$	0.5484	0.4934	0.5080	0.7270	0.5704	0.7368
$Q_1$, $P_3$, $P_2$	0.5200	0.4927	0.5402	0.8190	0.6278	0.8369
$Q_1$, $P_3$, $P_1$	0.4559	0.5028	0.5245	0.8198	0.6148	0.8351
$Q_1$, $P_2$, $P_1$	0.4061	0.4945	0.5376	0.8232	0.6662	0.8430
$P_3$, $P_2$, $P_1$	0.5371	0.5064	0.5123	0.5900	0.5703	0.6103
$Q_3$, $Q_2$, $Q_1$, $P_3$	0.6161	0.5157	0.5636	0.9135	0.6910	0.9226
$Q_3$, $Q_2$, $Q_1$, $P_2$	0.4501	0.4960	0.5432	0.9104	0.6906	0.9231
$Q_3$, $Q_2$, $Q_1$, $P_1$	0.4769	0.5116	0.5571	0.9108	0.6911	0.9306
$Q_3$, $Q_2$, $P_3$, $P_2$	0.3576	0.5001	0.5493	0.8481	0.6448	0.8642
$Q_3$, $Q_2$, $P_3$, $P_1$	0.5516	0.4927	0.5502	0.8423	0.6587	0.8596
$Q_3$, $Q_2$, $P_2$, $P_1$	0.4514	0.4995	0.5654	0.8485	0.6667	0.8652
$Q_3$, $Q_1$, $P_3$, $P_2$	0.4502	0.5018	0.5753	0.8920	0.6736	0.9052
$Q_3$, $Q_1$, $P_3$, $P_1$	0.5708	0.5029	0.5523	0.8986	0.6678	0.9065
$Q_3$, $Q_1$, $P_2$, $P_1$	0.6328	0.5003	0.5701	0.8957	0.6920	0.9107
$Q_3$, $P_3$, $P_2$, $P_1$	0.4682	0.4956	0.5519	0.8011	0.6825	0.8163
$Q_2$, $Q_1$, $P_3$, $P_2$	0.4894	0.4988	0.5346	0.8577	0.6144	0.8667
$Q_2$, $Q_1$, $P_3$, $P_1$	0.5418	0.4968	0.5179	0.8477	0.5928	0.8747
$Q_2$, $Q_1$, $P_2$, $P_1$	0.5582	0.4922	0.5202	0.8513	0.6274	0.8734
$Q_2$, $P_3$, $P_2$, $P_1$	0.5467	0.5073	0.5109	0.7137	0.5794	0.7443
$Q_1$, $P_3$, $P_2$, $P_1$	0.5075	0.5073	0.5237	0.8179	0.6029	0.8338
$Q_3$, $Q_2$, $Q_1$, $P_3$, $P_2$	0.5460	0.4992	0.5434	0.9110	0.6913	0.9201
$Q_3$, $Q_2$, $Q_1$, $P_3$, $P_1$	0.5520	0.4984	0.5477	0.9103	0.6909	0.9218
$Q_3$, $Q_2$, $Q_1$, $P_2$, $P_1$	0.5154	0.5028	0.5407	0.9084	0.6888	0.9281
$Q_3$, $Q_2$, $P_3$, $P_2$, $P_1$	0.4996	0.4843	0.5478	0.8377	0.6378	0.8638
$Q_3$, $Q_1$, $P_3$, $P_2$, $P_1$	0.4262	0.4940	0.5729	0.8862	0.6664	0.9089
$Q_2$, $Q_1$, $P_3$, $P_2$, $P_1$	0.5904	0.5024	0.5354	0.8485	0.6138	0.8732
$Q_3$, $Q_2$, $Q_1$, $P_3$, $P_2$, $P_1$	0.3930	0.5013	0.5597	0.9035	0.6729	0.9261

## AAA combination search results

The $F_1$ scores, sensitivities, and specificities achieved for AAA classification when using each of the six ML methods are shown in Tables 25, 26 and 27 respectively.

**Table 25:** The $F_1$ scores achieved across the combination search by each of the six classification methods

Input combination	Classification method					
	NB	LR	SVM	RF	MLP	GB
$Q_3$	0.4670	0.4881	0.8454	0.9095	0.8606	0.9294
$Q_2$	0.5754	0.4952	0.8246	0.9516	0.9092	0.9640
$Q_1$	0.4440	0.4843	0.9481	0.9741	0.9697	0.9805
$P_3$	0.4999	0.5102	0.8664	0.9027	0.8692	0.9226
$P_2$	0.5782	0.4944	0.8717	0.9087	0.8793	0.9311
$P_1$	0.4790	0.4826	0.8212	0.8771	0.8416	0.8884
$Q_3$, $Q_2$	0.3850	0.4983	0.8895	0.9753	0.9249	0.9843
$Q_3$, $Q_1$	0.4982	0.5029	0.9521	0.9840	0.9749	0.9919
$Q_3$, $P_3$	0.5126	0.4960	0.9215	0.9483	0.9249	0.9767
$Q_3$, $P_2$	0.6111	0.4958	0.9355	0.9543	0.9385	0.9770
$Q_3$, $P_1$	0.4737	0.4971	0.9286	0.9498	0.9448	0.9702
$Q_2$, $Q_1$	0.5523	0.4970	0.9523	0.9868	0.9718	0.9928
$Q_2$, $P_3$	0.5080	0.4994	0.9305	0.9604	0.9430	0.9805
$Q_2$, $P_2$	0.4756	0.4996	0.9371	0.9712	0.9552	0.9849
$Q_2$, $P_1$	0.4032	0.4975	0.9168	0.9689	0.9413	0.9828
$Q_1$, $P_3$	0.5350	0.5046	0.9630	0.9808	0.9741	0.9870
$Q_1$, $P_2$	0.4613	0.4981	0.9681	0.9820	0.9756	0.9900
$Q_1$, $P_1$	0.4909	0.5003	0.9747	0.9798	0.9801	0.9852
$P_3$, $P_2$	0.5343	0.5018	0.9247	0.9335	0.9305	0.9677
$P_3$, $P_1$	0.4857	0.5078	0.9321	0.9345	0.9311	0.9675
$P_2$, $P_1$	0.5431	0.5039	0.9213	0.9365	0.9405	0.9625
$Q_3$, $Q_2$, $Q_1$	0.4890	0.5164	0.9603	0.9912	0.9729	0.9962
$Q_3$, $Q_2$, $P_3$	0.5485	0.4993	0.9452	0.9771	0.9436	0.9905
$Q_3$, $Q_2$, $P_2$	0.5359	0.4998	0.9542	0.9791	0.9568	0.9910
$Q_3$, $Q_2$, $P_1$	0.4374	0.5070	0.9518	0.9803	0.9503	0.9906
$Q_3$, $Q_1$, $P_3$	0.5193	0.5085	0.9663	0.9861	0.9740	0.9936
$Q_3$, $Q_1$, $P_2$	0.5325	0.5034	0.9747	0.9884	0.9784	0.9939
$Q_3$, $Q_1$, $P_1$	0.4819	0.4943	0.9781	0.9850	0.9796	0.9936
$Q_3$, $P_3$, $P_2$	0.4106	0.4991	0.9479	0.9586	0.9434	0.9807
$Q_3$, $P_3$, $P_1$	0.4291	0.4901	0.9560	0.9598	0.9491	0.9846
$Q_3$, $P_2$, $P_1$	0.4537	0.4948	0.9492	0.9647	0.9515	0.9804
$Q_2$, $Q_1$, $P_3$	0.5071	0.5051	0.9685	0.9877	0.9795	0.9944
$Q_2$, $Q_1$, $P_2$	0.4853	0.4951	0.9724	0.9893	0.9797	0.9957
$Q_2$, $Q_1$, $P_1$	0.4459	0.4994	0.9752	0.9885	0.9816	0.9952
$Q_2$, $P_3$, $P_2$	0.4060	0.4932	0.9566	0.9714	0.9576	0.9873
$Q_2$, $P_3$, $P_1$	0.5857	0.4972	0.9577	0.9722	0.9582	0.9882
$Q_2$, $P_2$, $P_1$	0.4776	0.5030	0.9497	0.9755	0.9671	0.9892
$Q_1$, $P_3$, $P_2$	0.4224	0.4974	0.9729	0.9823	0.9788	0.9904
$Q_1$, $P_3$, $P_1$	0.4944	0.4987	0.9747	0.9813	0.9797	0.9897
$Q_1$, $P_2$, $P_1$	0.5362	0.5051	0.9756	0.9828	0.9827	0.9917
$P_3$, $P_2$, $P_1$	0.4406	0.5001	0.9479	0.9455	0.9517	0.9750
$Q_3$, $Q_2$, $Q_1$, $P_3$	0.5284	0.5135	0.9711	0.9914	0.9756	0.9965
$Q_3$, $Q_2$, $Q_1$, $P_2$	0.5279	0.5066	0.9784	0.9923	0.9794	0.9972
$Q_3$, $Q_2$, $Q_1$, $P_1$	0.4331	0.4983	0.9790	0.9903	0.9792	0.9961
$Q_3$, $Q_2$, $P_3$, $P_2$	0.5090	0.5041	0.9636	0.9797	0.9582	0.9930
$Q_3$, $Q_2$, $P_3$, $P_1$	0.5250	0.4963	0.9665	0.9784	0.9633	0.9922
$Q_3$, $Q_2$, $P_2$, $P_1$	0.4600	0.4887	0.9646	0.9829	0.9724	0.9937
$Q_3$, $Q_1$, $P_3$, $P_2$	0.4994	0.5003	0.9759	0.9880	0.9771	0.9939
$Q_3$, $Q_1$, $P_3$, $P_1$	0.5058	0.5060	0.9779	0.9867	0.9782	0.9942
$Q_3$, $Q_1$, $P_2$, $P_1$	0.4981	0.4974	0.9781	0.9869	0.9778	0.9950
$Q_3$, $P_3$, $P_2$, $P_1$	0.4679	0.5050	0.9634	0.9651	0.9595	0.9856
$Q_2$, $Q_1$, $P_3$, $P_2$	0.4910	0.4989	0.9776	0.9901	0.9759	0.9954
$Q_2$, $Q_1$, $P_3$, $P_1$	0.4893	0.5041	0.9794	0.9892	0.9772	0.9948
$Q_2$, $Q_1$, $P_2$, $P_1$	0.4849	0.4994	0.9771	0.9911	0.9800	0.9957
$Q_2$, $P_3$, $P_2$, $P_1$	0.4963	0.5081	0.9644	0.9748	0.9684	0.9903
$Q_1$, $P_3$, $P_2$, $P_1$	0.5090	0.5054	0.9763	0.9857	0.9788	0.9910
$Q_3$, $Q_2$, $Q_1$, $P_3$, $P_2$	0.4588	0.4997	0.9781	0.9915	0.9739	0.9970
$Q_3$, $Q_2$, $Q_1$, $P_3$, $P_1$	0.5224	0.4957	0.9800	0.9920	0.9767	0.9970
$Q_3$, $Q_2$, $Q_1$, $P_2$, $P_1$	0.5003	0.4947	0.9823	0.9912	0.9808	0.9966
$Q_3$, $Q_2$, $P_3$, $P_2$, $P_1$	0.4667	0.4900	0.9708	0.9828	0.9668	0.9948
$Q_3$, $Q_1$, $P_3$, $P_2$, $P_1$	0.5322	0.4962	0.9801	0.9874	0.9775	0.9938
$Q_2$, $Q_1$, $P_3$, $P_2$, $P_1$	0.4450	0.5064	0.9801	0.9892	0.9808	0.9961
$Q_3$, $Q_2$, $Q_1$, $P_3$, $P_2$, $P_1$	0.5083	0.4991	0.9820	0.9912	0.9785	0.9970

**Table 26:** The sensitivities achieved across the combination search by each of the six classification methods

Input combination	Classification method					
	NB	LR	SVM	RF	MLP	GB
$Q_3$	0.5683	0.5120	0.8568	0.8878	0.8661	0.9300
$Q_2$	0.5738	0.5089	0.8136	0.9355	0.9100	0.9638
$Q_1$	0.4451	0.4962	0.9517	0.9654	0.9673	0.9799
$P_3$	0.4846	0.5035	0.8785	0.8765	0.8660	0.9202
$P_2$	0.4451	0.5110	0.8712	0.9005	0.8818	0.9352
$P_1$	0.6616	0.4902	0.8491	0.8514	0.8308	0.8770
$Q_3$, $Q_2$	0.5927	0.4676	0.8868	0.9652	0.9308	0.9835
$Q_3$, $Q_1$	0.5541	0.5333	0.9508	0.9757	0.9747	0.9907
$Q_3$, $P_3$	0.4269	0.4894	0.9222	0.9282	0.9266	0.9746
$Q_3$, $P_2$	0.4746	0.5016	0.9325	0.9382	0.9379	0.9819
$Q_3$, $P_1$	0.5850	0.4760	0.9213	0.9317	0.9462	0.9694
$Q_2$, $Q_1$	0.2504	0.5034	0.9534	0.9810	0.9738	0.9919
$Q_2$, $P_3$	0.4111	0.4591	0.9285	0.9464	0.9439	0.9793
$Q_2$, $P_2$	0.5865	0.5093	0.9345	0.9604	0.9544	0.9836
$Q_2$, $P_1$	0.5669	0.4940	0.9227	0.9552	0.9471	0.9817
$Q_1$, $P_3$	0.4266	0.4741	0.9626	0.9729	0.9743	0.9850
$Q_1$, $P_2$	0.5075	0.4991	0.9664	0.9743	0.9780	0.9895
$Q_1$, $P_1$	0.5143	0.5055	0.9742	0.9715	0.9806	0.9841
$P_3$, $P_2$	0.4414	0.4981	0.9287	0.9209	0.9379	0.9673
$P_3$, $P_1$	0.5355	0.4956	0.9461	0.9109	0.9337	0.9631
$P_2$, $P_1$	0.4090	0.4957	0.9311	0.9260	0.9359	0.9596
$Q_3$, $Q_2$, $Q_1$	0.6548	0.5014	0.9592	0.9864	0.9760	0.9954
$Q_3$, $Q_2$, $P_3$	0.4363	0.4885	0.9445	0.9689	0.9482	0.9897
$Q_3$, $Q_2$, $P_2$	0.5720	0.5284	0.9506	0.9704	0.9620	0.9904
$Q_3$, $Q_2$, $P_1$	0.4962	0.5110	0.9455	0.9723	0.9511	0.9914
$Q_3$, $Q_1$, $P_3$	0.5329	0.4857	0.9666	0.9793	0.9774	0.9913
$Q_3$, $Q_1$, $P_2$	0.3570	0.4931	0.9701	0.9820	0.9794	0.9929
$Q_3$, $Q_1$, $P_1$	0.3667	0.5022	0.9771	0.9755	0.9805	0.9924
$Q_3$, $P_3$, $P_2$	0.6250	0.5064	0.9434	0.9445	0.9426	0.9822
$Q_3$, $P_3$, $P_1$	0.4716	0.4865	0.9564	0.9413	0.9473	0.9843
$Q_3$, $P_2$, $P_1$	0.5103	0.4982	0.9447	0.9522	0.9575	0.9819
$Q_2$, $Q_1$, $P_3$	0.4499	0.4986	0.9676	0.9815	0.9797	0.9933
$Q_2$, $Q_1$, $P_2$	0.6389	0.4936	0.9689	0.9838	0.9795	0.9947
$Q_2$, $Q_1$, $P_1$	0.6675	0.5043	0.9741	0.9817	0.9811	0.9945
$Q_2$, $P_3$, $P_2$	0.5890	0.4948	0.9564	0.9609	0.9598	0.9864
$Q_2$, $P_3$, $P_1$	0.4238	0.5033	0.9606	0.9619	0.9578	0.9868
$Q_2$, $P_2$, $P_1$	0.5582	0.5024	0.9540	0.9660	0.9686	0.9881
$Q_1$, $P_3$, $P_2$	0.5561	0.4904	0.9703	0.9736	0.9786	0.9898
$Q_1$, $P_3$, $P_1$	0.6229	0.5165	0.9753	0.9725	0.9799	0.9881
$Q_1$, $P_2$, $P_1$	0.4489	0.5084	0.9753	0.9750	0.9837	0.9896
$P_3$, $P_2$, $P_1$	0.6036	0.5139	0.9563	0.9278	0.9522	0.9726
$Q_3$, $Q_2$, $Q_1$, $P_3$	0.4318	0.5058	0.9684	0.9870	0.9803	0.9953
$Q_3$, $Q_2$, $Q_1$, $P_2$	0.5271	0.4841	0.9751	0.9879	0.9791	0.9959
$Q_3$, $Q_2$, $Q_1$, $P_1$	0.6257	0.4871	0.9768	0.9848	0.9794	0.9944
$Q_3$, $Q_2$, $P_3$, $P_2$	0.4330	0.5113	0.9615	0.9692	0.9620	0.9922
$Q_3$, $Q_2$, $P_3$, $P_1$	0.4955	0.4973	0.9639	0.9675	0.9661	0.9925
$Q_3$, $Q_2$, $P_2$, $P_1$	0.4783	0.4925	0.9610	0.9737	0.9660	0.9930
$Q_3$, $Q_1$, $P_3$, $P_2$	0.4914	0.4957	0.9741	0.9818	0.9795	0.9932
$Q_3$, $Q_1$, $P_3$, $P_1$	0.5768	0.5028	0.9778	0.9794	0.9788	0.9928
$Q_3$, $Q_1$, $P_2$, $P_1$	0.4613	0.4924	0.9749	0.9805	0.9771	0.9940
$Q_3$, $P_3$, $P_2$, $P_1$	0.6938	0.5114	0.9619	0.9516	0.9633	0.9856
$Q_2$, $Q_1$, $P_3$, $P_2$	0.5969	0.4915	0.9770	0.9861	0.9772	0.9944
$Q_2$, $Q_1$, $P_3$, $P_1$	0.5361	0.5044	0.9800	0.9846	0.9770	0.9938
$Q_2$, $Q_1$, $P_2$, $P_1$	0.5999	0.5042	0.9753	0.9867	0.9815	0.9944
$Q_2$, $P_3$, $P_2$, $P_1$	0.4892	0.4885	0.9676	0.9650	0.9693	0.9887
$Q_1$, $P_3$, $P_2$, $P_1$	0.3810	0.5027	0.9761	0.9790	0.9791	0.9887
$Q_3$, $Q_2$, $Q_1$, $P_3$, $P_2$	0.5180	0.5006	0.9749	0.9866	0.9752	0.9959
$Q_3$, $Q_2$, $Q_1$, $P_3$, $P_1$	0.4600	0.4811	0.9805	0.9873	0.9794	0.9963
$Q_3$, $Q_2$, $Q_1$, $P_2$, $P_1$	0.4965	0.5034	0.9824	0.9870	0.9808	0.9952
$Q_3$, $Q_2$, $P_3$, $P_2$, $P_1$	0.4020	0.5030	0.9704	0.9745	0.9692	0.9944
$Q_3$, $Q_1$, $P_3$, $P_2$, $P_1$	0.4284	0.5086	0.9809	0.9804	0.9763	0.9925
$Q_2$, $Q_1$, $P_3$, $P_2$, $P_1$	0.5795	0.4863	0.9812	0.9836	0.9811	0.9949
$Q_3$, $Q_2$, $Q_1$, $P_3$, $P_2$, $P_1$	0.4242	0.5024	0.9802	0.9861	0.9778	0.9959

**Table 27:** The specificities achieved across the combination search by each of the six classification methods

Input combination	Classification method					
	NB	LR	SVM	RF	MLP	GB
$Q_3$	0.4362	0.4805	0.8371	0.9276	0.8565	0.9290
$Q_2$	0.5761	0.4908	0.8324	0.9663	0.9087	0.9643
$Q_1$	0.4437	0.4806	0.9450	0.9825	0.9720	0.9811
$P_3$	0.5050	0.5126	0.8572	0.9244	0.8718	0.9248
$P_2$	0.6324	0.4890	0.8722	0.9156	0.8775	0.9277
$P_1$	0.4215	0.4803	0.8018	0.8972	0.8496	0.8976
$Q_3$, $Q_2$	0.3355	0.5086	0.8917	0.9851	0.9200	0.9851
$Q_3$, $Q_1$	0.4797	0.4927	0.9533	0.9922	0.9751	0.9931
$Q_3$, $P_3$	0.5422	0.4982	0.9210	0.9666	0.9235	0.9788
$Q_3$, $P_2$	0.6712	0.4939	0.9383	0.9691	0.9392	0.9724
$Q_3$, $P_1$	0.4392	0.5041	0.9351	0.9662	0.9436	0.97103
$Q_2$, $Q_1$	0.6675	0.4949	0.9514	0.9926	0.9701	0.9938
$Q_2$, $P_3$	0.5410	0.5129	0.9324	0.9735	0.9423	0.9817
$Q_2$, $P_2$	0.4411	0.4964	0.9394	0.9815	0.9561	0.9862
$Q_2$, $P_1$	0.3619	0.4987	0.9119	0.9819	0.9363	0.9840
$Q_1$, $P_3$	0.5747	0.5149	0.9635	0.9885	0.9741	0.9890
$Q_1$, $P_2$	0.4475	0.4979	0.9697	0.9896	0.9734	0.9906
$Q_1$, $P_1$	0.4834	0.4986	0.9753	0.9879	0.9797	0.9863
$P_3$, $P_2$	0.5682	0.5031	0.9213	0.9447	0.9241	0.9681
$P_3$, $P_1$	0.4698	0.5120	0.9199	0.9552	0.9289	0.9718
$P_2$, $P_1$	0.5932	0.5067	0.9130	0.9459	0.9446	0.9652
$Q_3$, $Q_2$, $Q_1$	0.4354	0.5217	0.9615	0.9961	0.9700	0.9970
$Q_3$, $Q_2$, $P_3$	0.5910	0.5030	0.9460	0.9850	0.9396	0.9914
$Q_3$, $Q_2$, $P_2$	0.5227	0.4904	0.9575	0.9876	0.9522	0.9917
$Q_3$, $Q_2$, $P_1$	0.4210	0.5057	0.9576	0.9880	0.9496	0.9899
$Q_3$, $Q_1$, $P_3$	0.5146	0.5163	0.9662	0.9928	0.9708	0.9960
$Q_3$, $Q_1$, $P_2$	0.5963	0.5069	0.9792	0.9947	0.9775	0.9950
$Q_3$, $Q_1$, $P_1$	0.5186	0.4918	0.9792	0.9944	0.9789	0.9949
$Q_3$, $P_3$, $P_2$	0.3553	0.4967	0.9520	0.9716	0.9442	0.9794
$Q_3$, $P_3$, $P_1$	0.4176	0.4913	0.9557	0.9769	0.9509	0.9850
$Q_3$, $P_2$, $P_1$	0.4371	0.4938	0.9533	0.9764	0.9461	0.9791
$Q_2$, $Q_1$, $P_3$	0.5266	0.5074	0.9695	0.9939	0.9794	0.9956
$Q_2$, $Q_1$, $P_2$	0.4362	0.4957	0.9758	0.9948	0.9799	0.9967
$Q_2$, $Q_1$, $P_1$	0.3824	0.4979	0.9764	0.9952	0.9822	0.9959
$Q_2$, $P_3$, $P_2$	0.3595	0.4928	0.9568	0.9814	0.9557	0.9882
$Q_2$, $P_3$, $P_1$	0.6529	0.4952	0.9552	0.9821	0.9586	0.9896
$Q_2$, $P_2$, $P_1$	0.4524	0.5033	0.9460	0.9847	0.9658	0.9903
$Q_1$, $P_3$, $P_2$	0.3867	0.4998	0.9754	0.9908	0.9791	0.9910
$Q_1$, $P_3$, $P_1$	0.4523	0.4929	0.9743	0.9898	0.9796	0.9913
$Q_1$, $P_2$, $P_1$	0.5683	0.5040	0.9759	0.9904	0.9819	0.9939
$P_3$, $P_2$, $P_1$	0.3946	0.4955	0.9405	0.9614	0.9513	0.9774
$Q_3$, $Q_2$, $Q_1$, $P_3$	0.5631	0.5162	0.9738	0.9958	0.9713	0.9977
$Q_3$, $Q_2$, $Q_1$, $P_2$	0.5282	0.5143	0.9816	0.9967	0.9797	0.9986
$Q_3$, $Q_2$, $Q_1$, $P_1$	0.3799	0.5021	0.9813	0.9958	0.9792	0.9978
$Q_3$, $Q_2$, $P_3$, $P_2$	0.5350	0.5018	0.9657	0.9899	0.9548	0.9939
$Q_3$, $Q_2$, $P_3$, $P_1$	0.5356	0.4960	0.9690	0.9890	0.9608	0.9921
$Q_3$, $Q_2$, $P_2$, $P_1$	0.4546	0.4875	0.9680	0.9918	0.9785	0.9944
$Q_3$, $Q_1$, $P_3$, $P_2$	0.5021	0.5019	0.9777	0.9941	0.9749	0.9947
$Q_3$, $Q_1$, $P_3$, $P_1$	0.4818	0.5072	0.9781	0.9939	0.9778	0.9956
$Q_3$, $Q_1$, $P_2$, $P_1$	0.5104	0.4991	0.9813	0.9932	0.9785	0.9961
$Q_3$, $P_3$, $P_2$, $P_1$	0.3990	0.5029	0.9648	0.9777	0.9561	0.9856
$Q_2$, $Q_1$, $P_3$, $P_2$	0.4566	0.5014	0.9782	0.9942	0.9748	0.9964
$Q_2$, $Q_1$, $P_3$, $P_1$	0.4742	0.5041	0.9789	0.9938	0.9774	0.9958
$Q_2$, $Q_1$, $P_2$, $P_1$	0.4481	0.4979	0.9790	0.9955	0.9786	0.9970
$Q_2$, $P_3$, $P_2$, $P_1$	0.4987	0.5149	0.9615	0.9843	0.9677	0.9919
$Q_1$, $P_3$, $P_2$, $P_1$	0.5527	0.5064	0.9765	0.9924	0.9787	0.9933
$Q_3$, $Q_2$, $Q_1$, $P_3$, $P_2$	0.4412	0.4995	0.9813	0.9965	0.9727	0.9981
$Q_3$, $Q_2$, $Q_1$, $P_3$, $P_1$	0.5446	0.5006	0.9796	0.9967	0.9743	0.9978
$Q_3$, $Q_2$, $Q_1$, $P_2$, $P_1$	0.5016	0.4919	0.9823	0.9955	0.9808	0.9981
$Q_3$, $Q_2$, $P_3$, $P_2$, $P_1$	0.4864	0.4858	0.9712	0.9910	0.9647	0.9952
$Q_3$, $Q_1$, $P_3$, $P_2$, $P_1$	0.5699	0.4922	0.9794	0.9944	0.9788	0.9951
$Q_2$, $Q_1$, $P_3$, $P_2$, $P_1$	0.4066	0.5133	0.9792	0.9947	0.9806	0.9973
$Q_3$, $Q_2$, $Q_1$, $P_3$, $P_2$, $P_1$	0.5370	0.4981	0.9839	0.9964	0.9792	0.9981

## AAA-L combination search results

The $F_1$ scores, sensitivities, and specificities achieved for AAA-L classification when employing the GB method are shown in Table 28.

**Table 28:** The $F_1$ scores, sensitivities and specificities achieved across the combination search by the GB method

Input combination	$F_1$	Sen.	Spec.
$Q_3$	0.8633	0.8561	0.8689
$Q_2$	0.9010	0.9103	0.8934
$Q_1$	0.9528	0.9630	0.9436
$P_3$	0.8305	0.8383	0.8250
$P_2$	0.8380	0.8529	0.8274
$P_1$	0.8005	0.7700	0.8209
$Q_3$, $Q_2$	0.9387	0.9390	0.9385
$Q_3$, $Q_1$	0.9683	0.9681	0.9685
$Q_3$, $P_3$	0.9045	0.8968	0.9109
$Q_3$, $P_2$	0.9151	0.9127	0.9172
$Q_3$, $P_1$	0.8989	0.8942	0.9028
$Q_2$, $Q_1$	0.9711	0.9741	0.9683
$Q_2$, $P_3$	0.9176	0.9256	0.9109
$Q_2$, $P_2$	0.9229	0.9328	0.9145
$Q_2$, $P_1$	0.9234	0.9258	0.9215
$Q_1$, $P_3$	0.9569	0.9558	0.9580
$Q_1$, $P_2$	0.9606	0.9645	0.9570
$Q_1$, $P_1$	0.9618	0.9609	0.9628
$P_3$, $P_2$	0.8852	0.8889	0.8824
$P_3$, $P_1$	0.8877	0.8889	0.8869
$P_2$, $P_1$	0.884	0.8858	0.8838
$Q_3$, $Q_2$, $Q_1$	0.9777	0.9788	0.9767
$Q_3$, $Q_2$, $P_3$	0.9454	0.9513	0.9402
$Q_3$, $Q_2$, $P_2$	0.9455	0.9498	0.9417
$Q_3$, $Q_2$, $P_1$	0.9481	0.9537	0.9431
$Q_3$, $Q_1$, $P_3$	0.9693	0.9743	0.9647
$Q_3$, $Q_1$, $P_2$	0.9695	0.9748	0.9647
$Q_3$, $Q_1$, $P_1$	0.9668	0.9642	0.9693
$Q_3$, $P_3$, $P_2$	0.9148	0.9105	0.9186
$Q_3$, $P_3$, $P_1$	0.9178	0.9232	0.9133
$Q_3$, $P_2$, $P_1$	0.9217	0.9163	0.9265
$Q_2$, $Q_1$, $P_3$	0.9770	0.9788	0.9753
$Q_2$, $Q_1$, $P_2$	0.9715	0.9729	0.9702
$Q_2$, $Q_1$, $P_1$	0.9737	0.9762	0.9714
$Q_2$, $P_3$, $P_2$	0.9327	0.9434	0.9234
$Q_2$, $P_3$, $P_1$	0.9285	0.9299	0.9273
$Q_2$, $P_2$, $P_1$	0.9345	0.9304	0.9381
$Q_1$, $P_3$, $P_2$	0.9606	0.9640	0.9575
$Q_1$, $P_3$, $P_1$	0.9637	0.9676	0.9601
$Q_1$, $P_2$, $P_1$	0.9607	0.9625	0.9592
$P_3$, $P_2$, $P_1$	0.8996	0.9038	0.8963
$Q_3$, $Q_2$, $Q_1$, $P_3$	0.9767	0.9781	0.9755
$Q_3$, $Q_2$, $Q_1$, $P_2$	0.9788	0.9786	0.9791
$Q_3$, $Q_2$, $Q_1$, $P_1$	0.9759	0.9791	0.9729
$Q_3$, $Q_2$, $P_3$, $P_2$	0.9484	0.9510	0.9462
$Q_3$, $Q_2$, $P_3$, $P_1$	0.9487	0.9525	0.9453
$Q_3$, $Q_2$, $P_2$, $P_1$	0.9472	0.9529	0.9421
$Q_3$, $Q_1$, $P_3$, $P_2$	0.9670	0.9654	0.9685
$Q_3$, $Q_1$, $P_3$, $P_1$	0.9673	0.9678	0.9669
$Q_3$, $Q_1$, $P_2$, $P_1$	0.9704	0.9683	0.9724
$Q_3$, $P_3$, $P_2$, $P_1$	0.9217	0.9227	0.9210
$Q_2$, $Q_1$, $P_3$, $P_2$	0.9754	0.9781	0.9729
$Q_2$, $Q_1$, $P_3$, $P_1$	0.9774	0.9784	0.9765
$Q_2$, $Q_1$, $P_2$, $P_1$	0.9772	0.9776	0.9770
$Q_2$, $P_3$, $P_2$, $P_1$	0.9352	0.9436	0.9280
$Q_1$, $P_3$, $P_2$, $P_1$	0.9587	0.9659	0.9522
$Q_3$, $Q_2$, $Q_1$, $P_3$, $P_2$	0.9744	0.9731	0.9758
$Q_3$, $Q_2$, $Q_1$, $P_3$, $P_1$	0.9820	0.9834	0.9808
$Q_3$, $Q_2$, $Q_1$, $P_2$, $P_1$	0.9802	0.9796	0.9808
$Q_3$, $Q_2$, $P_3$, $P_2$, $P_1$	0.9513	0.9541	0.9489
$Q_3$, $Q_1$, $P_3$, $P_2$, $P_1$	0.9725	0.9712	0.9738
$Q_2$, $Q_1$, $P_3$, $P_2$, $P_1$	0.9757	0.9815	0.9702
$Q_3$, $Q_2$, $Q_1$, $P_3$, $P_2$, $P_1$	0.9809	0.9808	0.9810

## GB results for all disease forms

The $F_1$ scores achieved for all forms of disease classification (including AAA-L) when providing each combination of input measurements are shown when employing the GB method in Fig. 21.
